# Supplementary material for: FlowMap: Geometry–Dynamics Consistent Embedding of RNA Velocity for Interpretable Cellular Trajectories
Source: bioRxiv. 2026 Sep 22:2026.09.21.753192. Preprint. [Version 1] doi: 10.64898/2026.09.21.753192 (PMC13622152; doi:10.64898/2026.09.21.753192)
Supplement: Supplementary file 1 [file NIHPP2026.09.21.753192v1-supplement-2.pdf]

bioRxiv preprint doi: https://doi.org/10.64898/2026.09.21.753192; this version posted September 24, 2026. The copyright holder for this preprint (which was not certified by peer review) is the author/funder, who has granted bioRxiv a license to display the preprint in perpetuity. It is made available under a CC-BY 4.0 International license.

Supplementary Tables

Capability	FlowMap	Dynamo	scVelo	Velocyto
**Velocity Modeling**				
Velocity estimation	×	✓	✓	✓
Kinetic parameter estimation	×	✓	✓	×
**Embedding Construction**				
Differentiable manifold	✓	✓*	×	×
Embedding reconstruction & denoising	✓	×	×	×
Embedding quality evaluation	✓	×	×	×
**Dynamical Analysis**				
Gene expression–visualization linkage	✓	×	×	×
Marker identification from embedding	✓	×	×	×
Pseudotime	✓	✓	✓	×
Least Action Paths (LAP)	✓	✓	×	×
Fixed-point identification	✓	✓	×	×
**Gene Regulatory Inference**				
Regulation analysis	×	✓	×	×
Perturbation simulation	×	✓	×	×

Table S1: **Comparison of capabilities across RNA velocity and dynamical modeling frameworks.** FlowMap focuses on geometry-aware embedding construction and downstream dynamical analysis, while Dynamo, scVelo, and Velocyto emphasize velocity and kinetic parameter estimation. *Dynamo models a differentiable vector field in gene or PCA space, but does not define a differentiable embedding manifold.

Supplementary Figures

bioRxiv preprint doi: https://doi.org/10.64898/2026.09.21.753192; this version posted September 24, 2026. The copyright holder for this preprint (which was not certified by peer review) is the author/funder, who has granted bioRxiv a license to display the preprint in perpetuity. It is made available under a CC-BY 4.0 International license.

Dataset	α	Jaccard Similarity	Trust-worthiness	Magnitude Correlation	Cosine Similarity	Smoothness
straight_line	0.0	0.0557	0.8609	–	0.9869	**0.9787**
	0.5	0.0602	0.8650	–	**0.9908**	0.9771
	1.0	0.0601	0.8646	–	0.9824	0.9769
	2.0	**0.0614**	**0.8654**	–	0.9853	0.9756
sine_curve	0.0	0.0584	0.8646	0.0004	**0.9867**	**0.9761**
	0.5	0.0604	0.8674	0.0045	0.9771	0.9759
	1.0	0.0592	**0.8675**	0.0150	0.9841	0.9758
	2.0	**0.0598**	0.8663	**0.0214**	0.9813	**0.9761**
branch_2	0.0	0.0398	0.8724	0.4888	0.9666	0.9818
	0.5	**0.0420**	**0.8739**	0.5314	0.9731	0.9826
	1.0	0.0409	0.8736	0.5470	0.9763	0.9839
	2.0	0.0412	0.8731	**0.5704**	**0.9772**	**0.9855**
branch_4	0.0	0.0310	0.8914	0.6334	0.9754	0.9889
	0.5	0.0317	**0.8928**	0.6738	**0.9793**	**0.9893**
	1.0	0.0313	0.8922	**0.6861**	0.9773	0.9886
	2.0	**0.0321**	0.8923	0.6855	0.9779	0.9890
rotation	0.0	0.0658	0.8303	0.3953	0.7320	0.9491
	0.5	0.0668	0.8338	0.3877	0.7387	**0.9517**
	1.0	0.0678	0.8363	**0.4050**	0.7415	0.9506
	2.0	**0.0682**	**0.8370**	0.3919	**0.7438**	0.9512
spiral	0.0	0.0704	0.8202	0.4260	**0.6888**	0.9400
	0.5	**0.0722**	**0.8265**	0.4504	0.6855	0.9421
	1.0	0.0709	0.8250	0.4530	0.6826	**0.9424**
	2.0	0.0718	0.8249	**0.4700**	0.6804	0.9420
saddle	0.0	0.0846	0.8473	**0.3554**	0.7081	**0.9362**
	0.5	0.0883	0.8526	0.3112	0.7313	0.9352
	1.0	0.0889	**0.8531**	0.3010	0.7334	0.9354
	2.0	**0.0891**	0.8519	0.2857	**0.7362**	0.9332
quadratic_source_sink	0.0	0.0713	0.8237	0.5240	0.7350	0.9286
	0.5	**0.0762**	**0.8288**	0.5627	0.7665	0.9303
	1.0	0.0742	0.8275	0.5518	0.7726	**0.9313**
	2.0	0.0750	0.8276	**0.5634**	**0.7814**	0.9283

Table S2: **Effect of phase-distance weighting on FlowMap embeddings.** Simulation benchmark across synthetic dynamical systems using different phase-distance weighting strengths α. Bold indicates the best metric value across α for each dataset.

bioRxiv preprint doi: https://doi.org/10.64898/2026.09.21.753192; this version posted September 24, 2026. The copyright holder for this preprint (which was not certified by peer review) is the author/funder, who has granted bioRxiv a license to display the preprint in perpetuity. It is made available under a CC-BY 4.0 International license.

Dataset	Method	Jaccard	Trustworthiness	Magnitude Corr.	Cosine Sim.	Smoothness
Straight Line	FlowMap	**0.0894**	**0.9150**	–	**0.9846**	**0.9805**
	GraphVelo	0.0576	0.8618	–	0.9076	0.8638
	scVelo	0.0596	0.8634	–	0.9044	0.9079
	Dynamo	0.0598	0.8653	–	0.8587	0.8765
	VeloViz	0.0178	0.5862	–	0.0734	0.6663
Sine Curve	FlowMap	**0.0847**	**0.9138**	0.0250	**0.9882**	**0.9818**
	GraphVelo	0.0570	0.8646	**0.0457**	0.8860	0.8413
	scVelo	0.0588	0.8653	0.0023	0.8853	0.9006
	Dynamo	0.0591	0.8652	0.0161	0.8386	0.8667
	VeloViz	0.0249	0.6507	-0.0279	0.3644	0.7062
Branch 2	FlowMap	**0.0602**	**0.9160**	**0.5958**	**0.9822**	**0.9870**
	GraphVelo	0.0372	0.8690	0.4029	0.8328	0.8224
	scVelo	0.0417	0.8728	0.1530	0.8060	0.8660
	Dynamo	0.0421	0.8727	0.0696	0.7690	0.8457
	VeloViz	0.0153	0.6028	-0.3201	0.1518	0.6632
Branch 4	FlowMap	**0.0484**	**0.9281**	**0.6961**	**0.9828**	**0.9917**
	GraphVelo	0.0321	0.8919	0.4981	0.8357	0.8275
	scVelo	0.0318	0.8917	0.2902	0.8119	0.8697
	Dynamo	0.0316	0.8917	0.1317	0.7841	0.8518
	VeloViz	0.0082	0.6433	-0.2253	0.2293	0.6789
Rotation	FlowMap	**0.1146**	**0.9094**	**0.5353**	**0.8739**	**0.9629**
	GraphVelo	0.0700	0.8342	0.3584	0.7757	0.7643
	scVelo	0.0674	0.8317	0.1817	0.7207	0.8203
	Dynamo	0.0672	0.8325	0.0889	0.7014	0.8070
	VeloViz	0.0425	0.7354	0.3070	0.5944	0.7623
Spiral	FlowMap	**0.1220**	**0.9058**	**0.6176**	**0.8251**	**0.9508**
	GraphVelo	0.0697	0.8177	0.4869	0.7816	0.7601
	scVelo	0.0744	0.8264	-0.0310	0.7339	0.7907
	Dynamo	0.0733	0.8258	-0.0738	0.6903	0.7795
	VeloViz	0.0732	0.8309	0.1919	0.5851	0.7607
Saddle	FlowMap	**0.1412**	**0.9196**	**0.3656**	**0.8379**	**0.9391**
	GraphVelo	0.0831	0.8526	0.3599	0.7235	0.7599
	scVelo	0.0872	0.8518	-0.1183	0.6382	0.7787
	Dynamo	0.0856	0.8519	-0.2107	0.6470	0.7831
	VeloViz	0.0404	0.6790	-0.1663	0.3891	0.7565
Quadratic Source/Sink	FlowMap	**0.1138**	**0.8948**	**0.6529**	0.7752	**0.9309**
	GraphVelo	0.0691	0.8156	-0.0852	**0.8885**	0.7495
	scVelo	0.0754	0.8231	0.2197	0.8684	0.8176
	Dynamo	0.0750	0.8232	0.2004	0.8312	0.8007
	VeloViz	0.0568	0.6549	0.2465	0.5141	0.6898

Table S3: Benchmarking results on simulated dynamical systems. Bold indicates the best-performing method for each metric within each dataset.

bioRxiv preprint doi: https://doi.org/10.64898/2026.09.21.753192; this version posted September 24, 2026. The copyright holder for this preprint (which was not certified by peer review) is the author/funder, who has granted bioRxiv a license to display the preprint in perpetuity. It is made available under a CC-BY 4.0 International license.

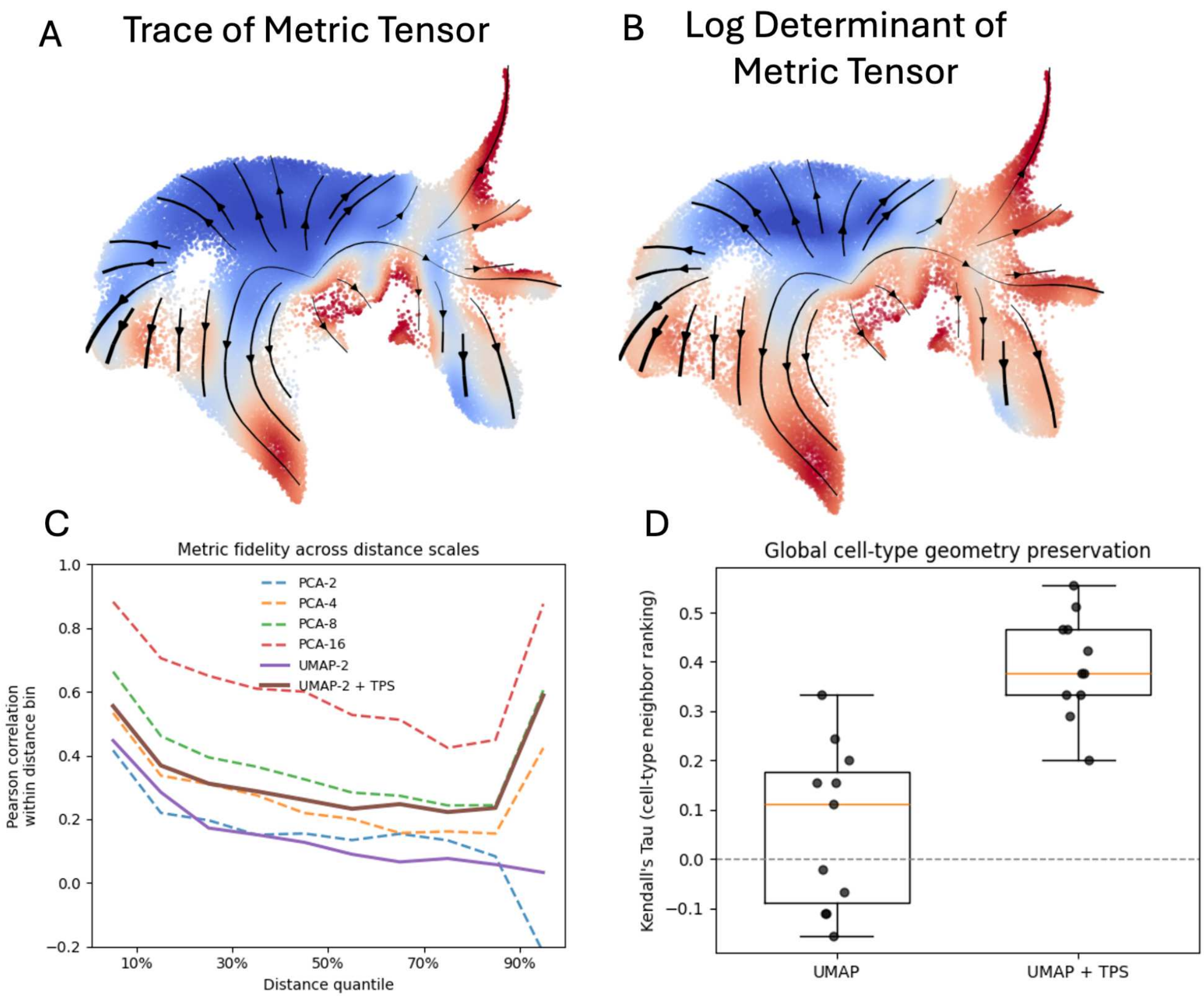

Figure S1: **Geometry-aware correction of 2D embeddings using TPS.** (A,B) Scalar summaries of the induced metric tensor on the embedding, visualized as the trace (A) and log-determinant (B). These quantities highlight spatially heterogeneous stretching and compression across the embedding, indicating that geometric distortion is not uniform. (C) Metric fidelity across distance scales. For each cell, pairwise distances to all other cells were computed in the ambient space and in each embedding. Distances were binned by ambient-space distance quantiles, and Pearson correlation between ambient and embedded distances was computed within each bin. UMAP exhibits rapid degradation of distance fidelity beyond short ranges, whereas UMAP+TPS improves correlations across intermediate and long distances, approaching the performance of low-dimensional PCA (PCA-4 to PCA-8). (D) Global cell-type geometry preservation measured by Kendall's Tau correlation of cell-type neighbor rankings, following Chari and Pachter. TPS correction substantially improves preservation of global cell-type relationships relative to UMAP alone.

bioRxiv preprint doi: https://doi.org/10.64898/2026.09.21.753192; this version posted September 24, 2026. The copyright holder for this preprint (which was not certified by peer review) is the author/funder, who has granted bioRxiv a license to display the preprint in perpetuity. It is made available under a CC-BY 4.0 International license.

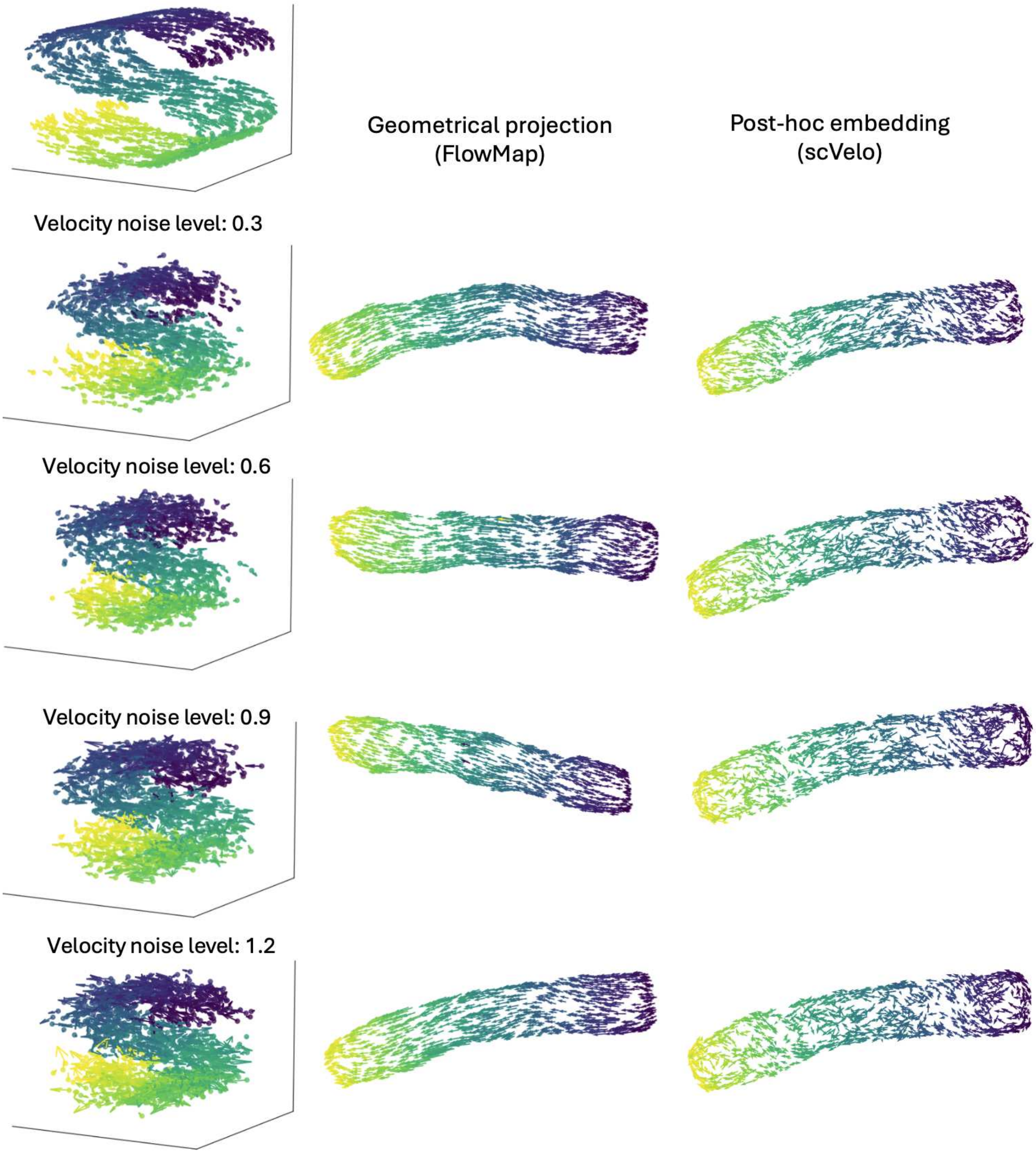

Figure S2: **Geometric projection is necessary for stable velocity visualization under noise.** *Left:* Ground-truth three-dimensional S-curve with increasing levels of additive velocity noise (top to bottom), colored by latent phase. *Middle (FlowMap):* Velocity vectors projected onto the learned manifold using a geometric (tangent-space) constraint, yielding coherent and stable flows even at high noise levels. *Right (scVelo):* Post-hoc velocity embedding obtained without enforcing geometric consistency, resulting in distorted and unstable vector fields as noise increases.

bioRxiv preprint doi: https://doi.org/10.64898/2026.09.21.753192; this version posted September 24, 2026. The copyright holder for this preprint (which was not certified by peer review) is the author/funder, who has granted bioRxiv a license to display the preprint in perpetuity. It is made available under a CC-BY 4.0 International license.

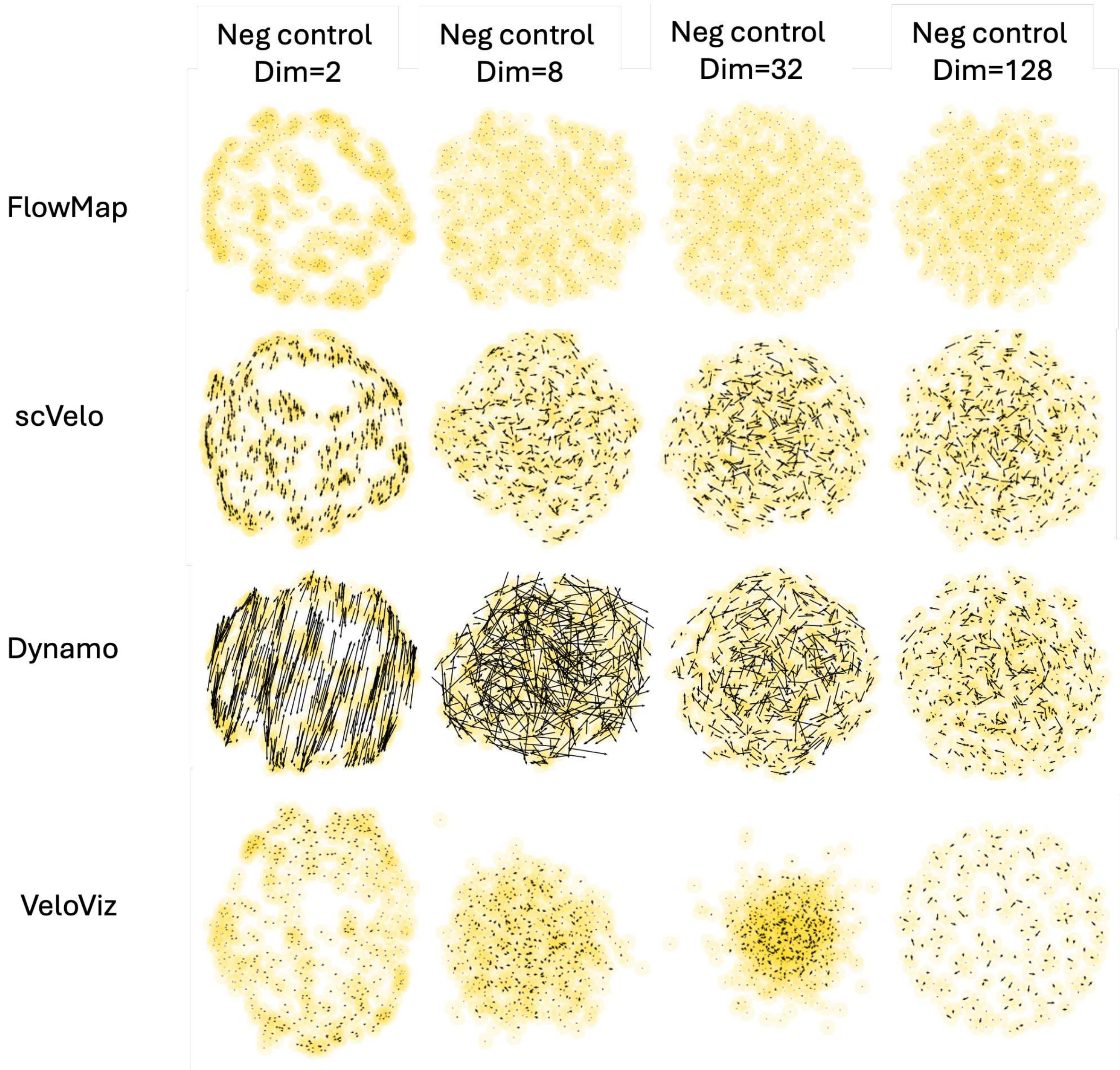

Figure S3: **Negative control for embedding-induced velocity artifacts.** RNA velocity vectors were generated as independent Gaussian noise in the original expression space at varying input dimensionalities (columns: Dim = 2, 8, 32, 128). Cells were embedded into two dimensions and velocities were visualized using four embedding strategies (rows). Despite the absence of any underlying dynamical structure, neighborhood-based embedding approaches produce visually coherent flow patterns that depend on the embedding geometry and smoothing procedure. In contrast, geometry-constrained projection suppresses these spurious patterns, yielding near-zero embedded velocities consistent with the absence of true dynamics. This illustrates that apparent low-dimensional flow structure can arise purely from embedding and interpolation effects, and that enforcing geometric consistency mitigates such artifacts.

bioRxiv preprint doi: https://doi.org/10.64898/2026.09.21.753192; this version posted September 24, 2026. The copyright holder for this preprint (which was not certified by peer review) is the author/funder, who has granted bioRxiv a license to display the preprint in perpetuity. It is made available under a CC-BY 4.0 International license.

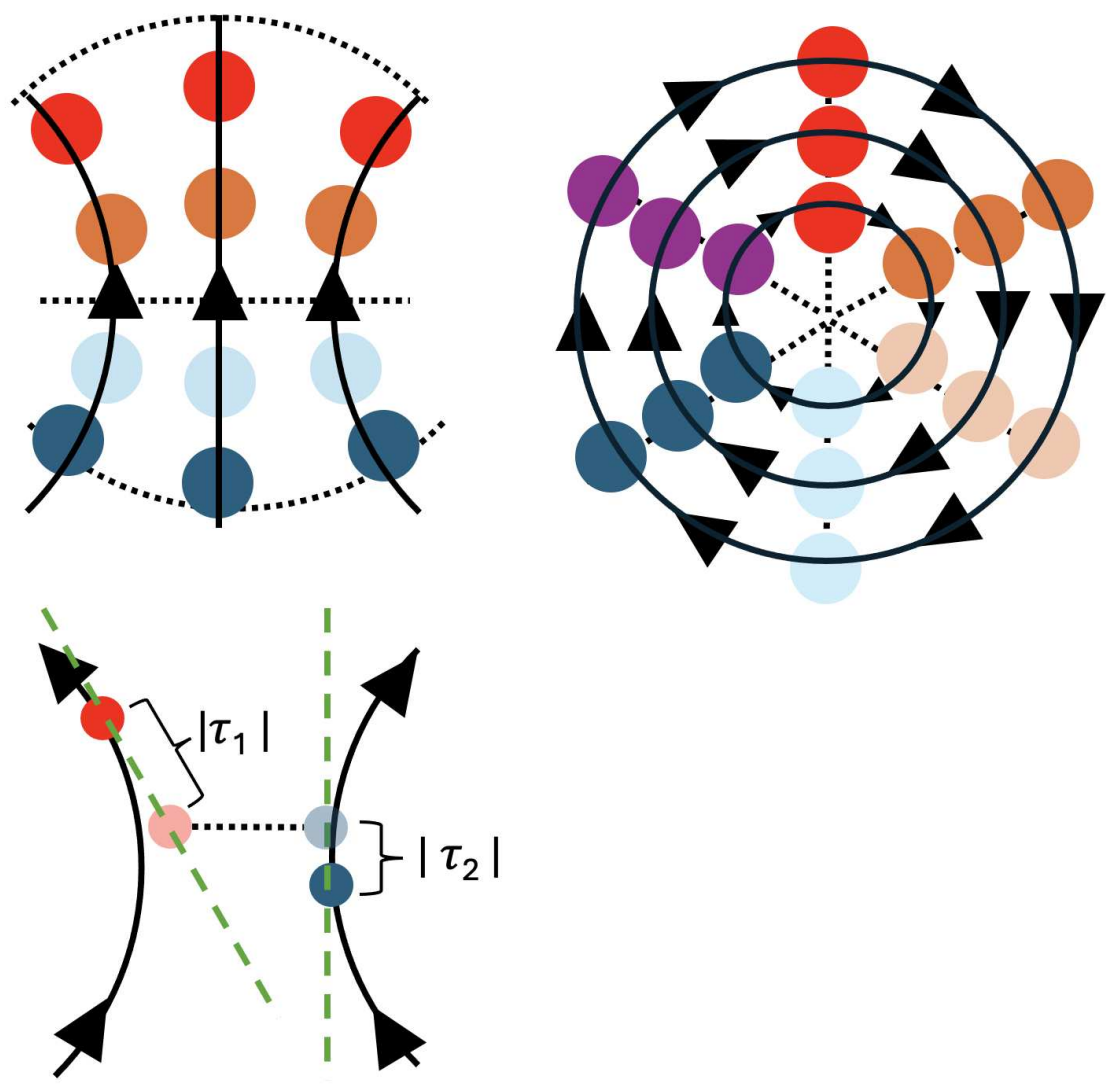

$$\tau_1, \tau_2 = argmin_{(\tau_1,\tau_2)} (x_1 + \tau_1 v_1 - x_2 - \tau_2 v_2)^2$$
$$s.t \quad \tau_1^2 + \tau_2^2 \le c^2$$
$$D_{ij}^2 = (\tau_1 - \tau_2)^2$$

Figure S4: **Conceptual illustration of phase along RNA velocity flow.** Three examples of RNA velocity fields with labeled phase. Cells with the same color are at the same phase and lie on curves perpendicular to the local velocity field, representing equivalent positions along a continuous trajectory. Although such cells may differ in expression, they are synchronized in their progression along the flow. For nearby cells, phase is estimated by allowing each cell to move along its velocity direction and solving for the relative offsets (τ_1, τ_2) that best align their expression states. The resulting phase distance $D_{ij}^2 = (\tau_1 - \tau_2)^2$ captures separation along the flow direction rather than transcriptional similarity alone.

bioRxiv preprint doi: https://doi.org/10.64898/2026.09.21.753192; this version posted September 24, 2026. The copyright holder for this preprint (which was not certified by peer review) is the author/funder, who has granted bioRxiv a license to display the preprint in perpetuity. It is made available under a CC-BY 4.0 International license.

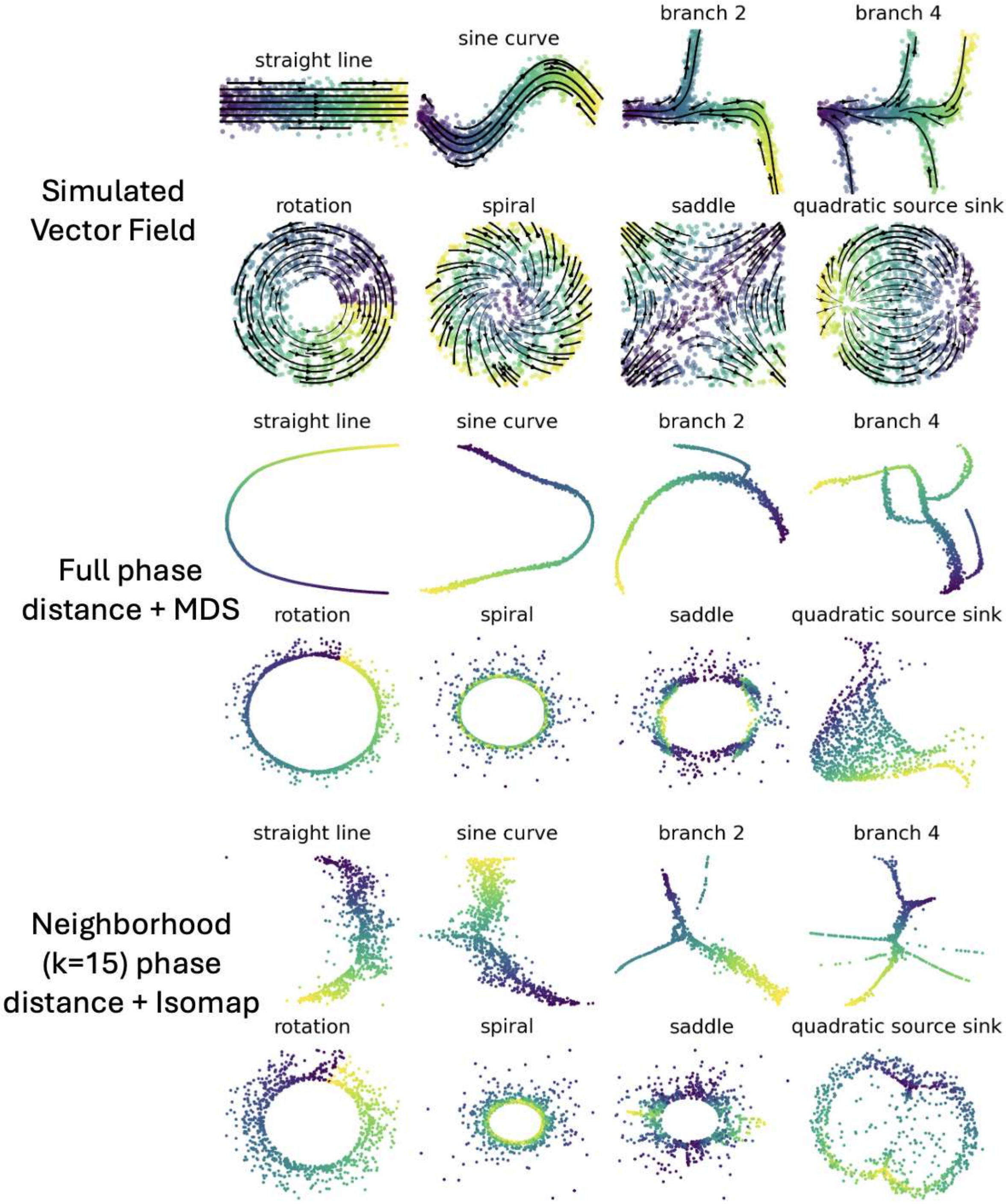

Figure S5: **Phase distance captures cellular position along flow curves in simulated vector fields. Top:** Simulated vector fields used for benchmarking, including linear, nonlinear, branching, rotational, spiral, saddle, and source–sink dynamics. Points are colored by true temporal position along the flow. **Middle:** Embeddings obtained using the full pairwise phase distance matrix followed by multidimensional scaling (MDS). Phase distance alone is sufficient to recover coherent low-dimensional representations that reflect progression along flow curves, straightening trajectories and separating branches according to their dynamical ordering. **Bottom:** Embeddings constructed using neighborhood-based phase distance ($k = 15$) and an Isomap-style procedure, where global distances are computed as shortest paths on the phase-distance graph.

bioRxiv preprint doi: https://doi.org/10.64898/2026.09.21.753192; this version posted September 24, 2026. The copyright holder for this preprint (which was not certified by peer review) is the author/funder, who has granted bioRxiv a license to display the preprint in perpetuity. It is made available under a CC-BY 4.0 International license.

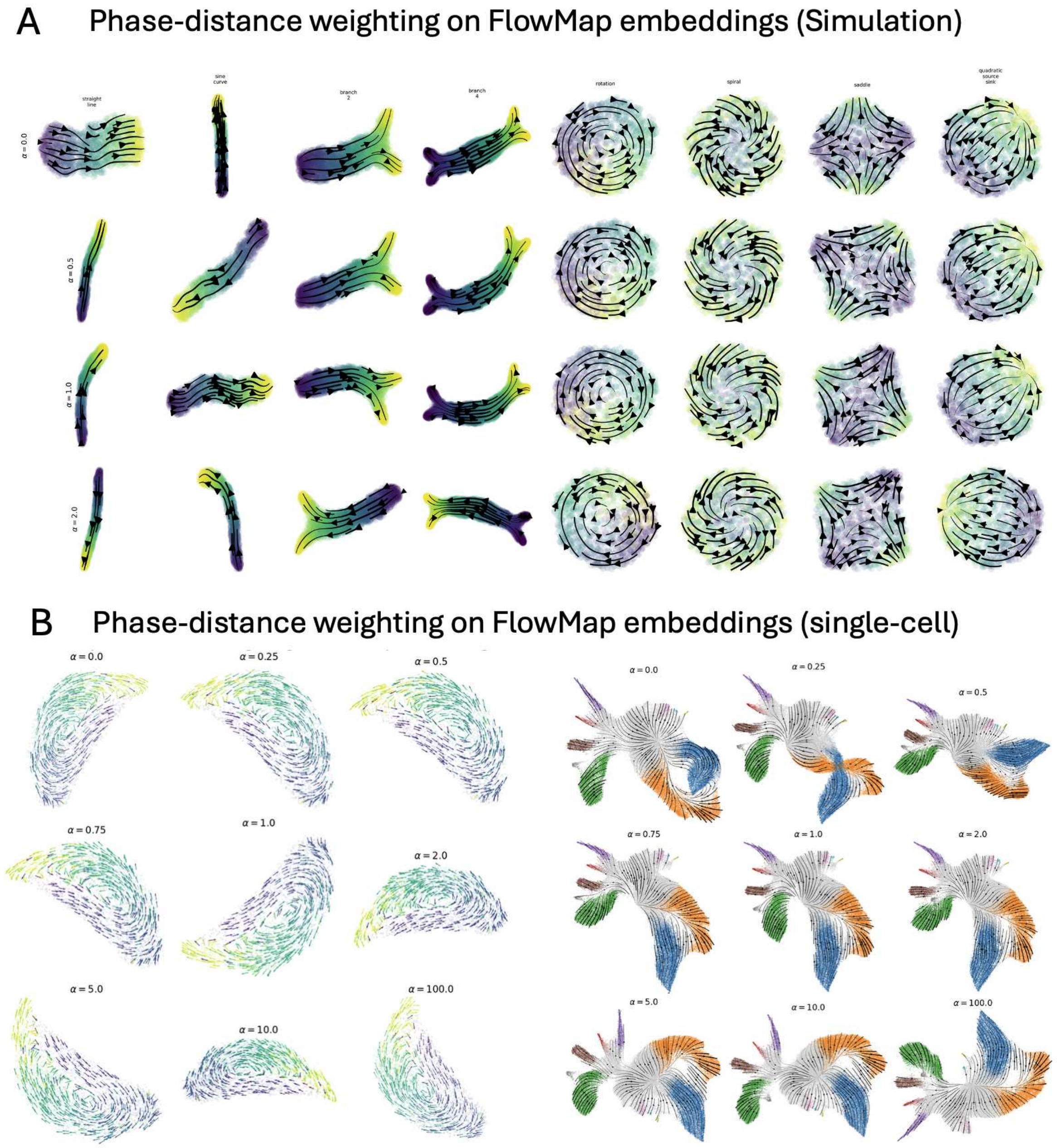

Figure S6: **Phase-distance weighting on FlowMap embeddings.** (A) Simulated vector field benchmark across eight synthetic dynamical systems. Rows correspond to increasing phase-distance weighting strength α, where the embedding distance is defined as Euclidean distance plus an additional phase-distance term. (B) Phase-distance weighting on real single-cell datasets. Left: cell-cycle dynamics. Right: hematopoietic differentiation in the LARRY dataset.

bioRxiv preprint doi: https://doi.org/10.64898/2026.09.21.753192; this version posted September 24, 2026. The copyright holder for this preprint (which was not certified by peer review) is the author/funder, who has granted bioRxiv a license to display the preprint in perpetuity. It is made available under a CC-BY 4.0 International license.

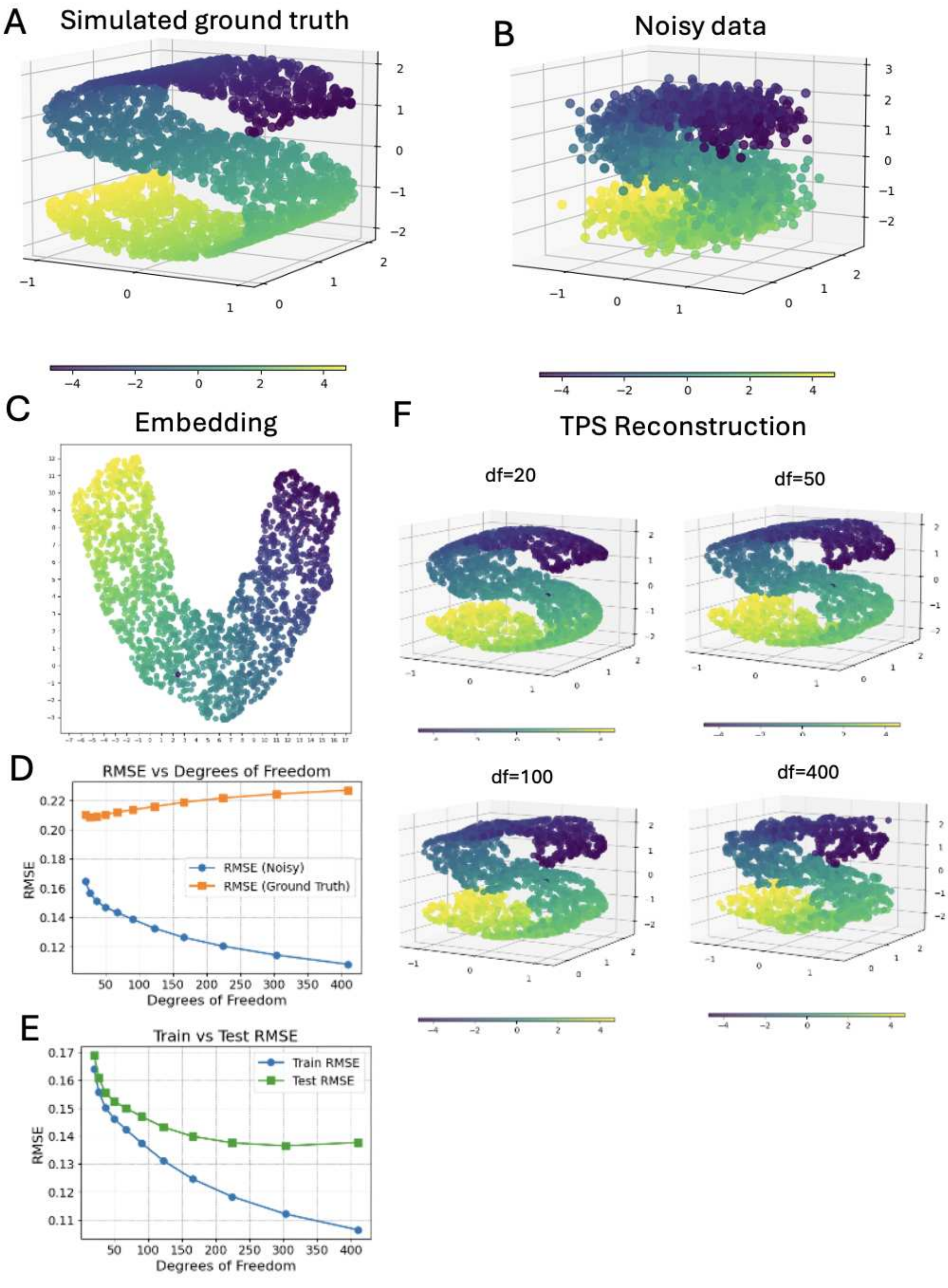

Figure S7: **Thin-plate spline (TPS) surface reconstruction with varying degrees of freedom.** (A) Simulated ground-truth manifold. (B) Noisy observations sampled from the same manifold. (C) Low-dimensional embedding constructed from the noisy data. (D) Reconstruction RMSE as a function of TPS degrees of freedom, evaluated against noisy data and ground truth. (E) Train and test RMSE as a function of degrees of freedom. (F) TPS reconstructions of the manifold for different degrees of freedom.

bioRxiv preprint doi: https://doi.org/10.64898/2026.09.21.753192; this version posted September 24, 2026. The copyright holder for this preprint (which was not certified by peer review) is the author/funder, who has granted bioRxiv a license to display the preprint in perpetuity. It is made available under a CC-BY 4.0 International license.

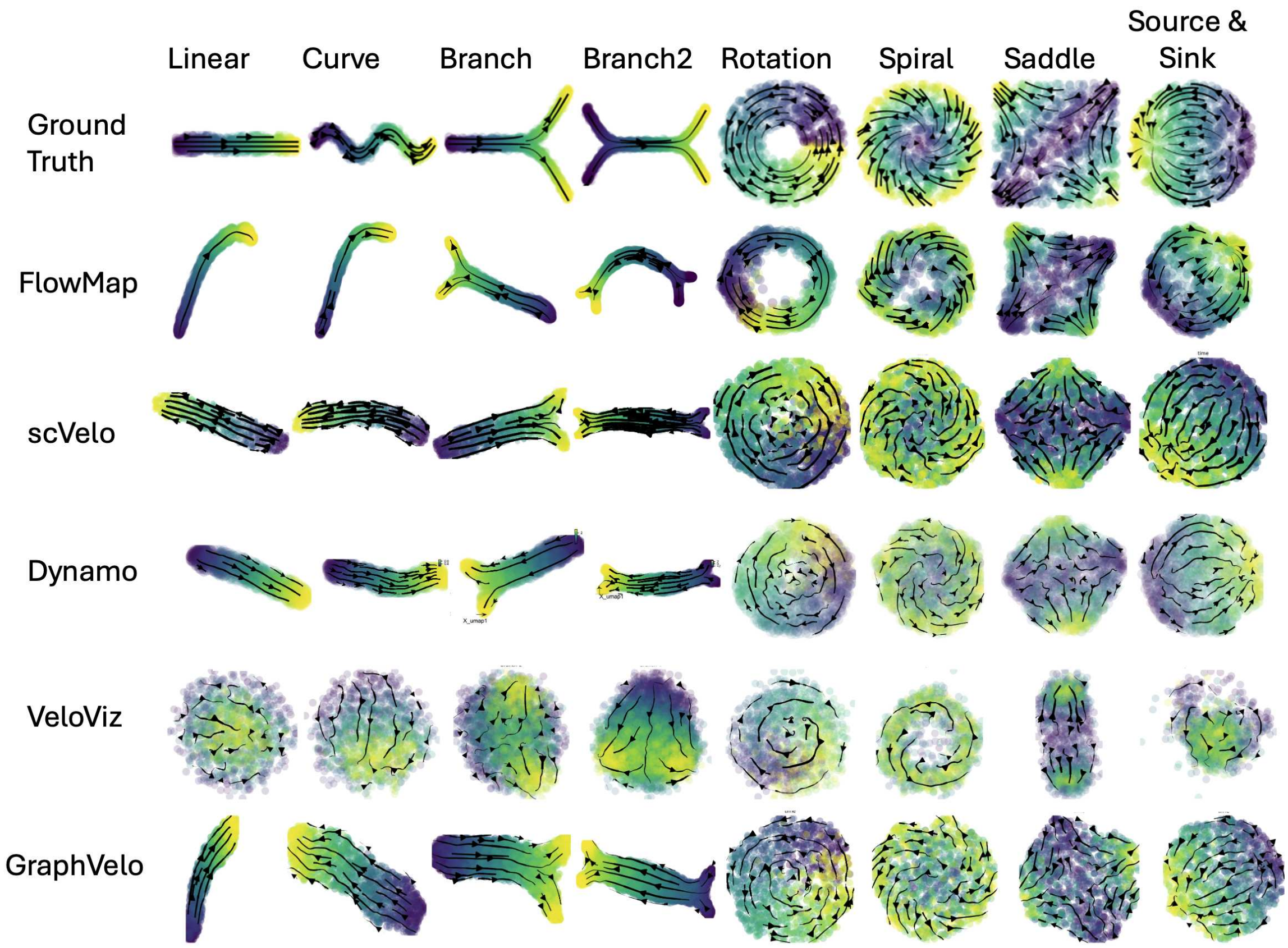

Figure S8: **Simulation benchmark of FlowMap across diverse dynamical systems.** Synthetic vector fields with linear, branching, and cyclic dynamics were used to evaluate embedding and velocity recovery. Cells are colored by ground-truth trajectory position, with arrows indicating velocity.

bioRxiv preprint doi: https://doi.org/10.64898/2026.09.21.753192; this version posted September 24, 2026. The copyright holder for this preprint (which was not certified by peer review) is the author/funder, who has granted bioRxiv a license to display the preprint in perpetuity. It is made available under a CC-BY 4.0 International license.

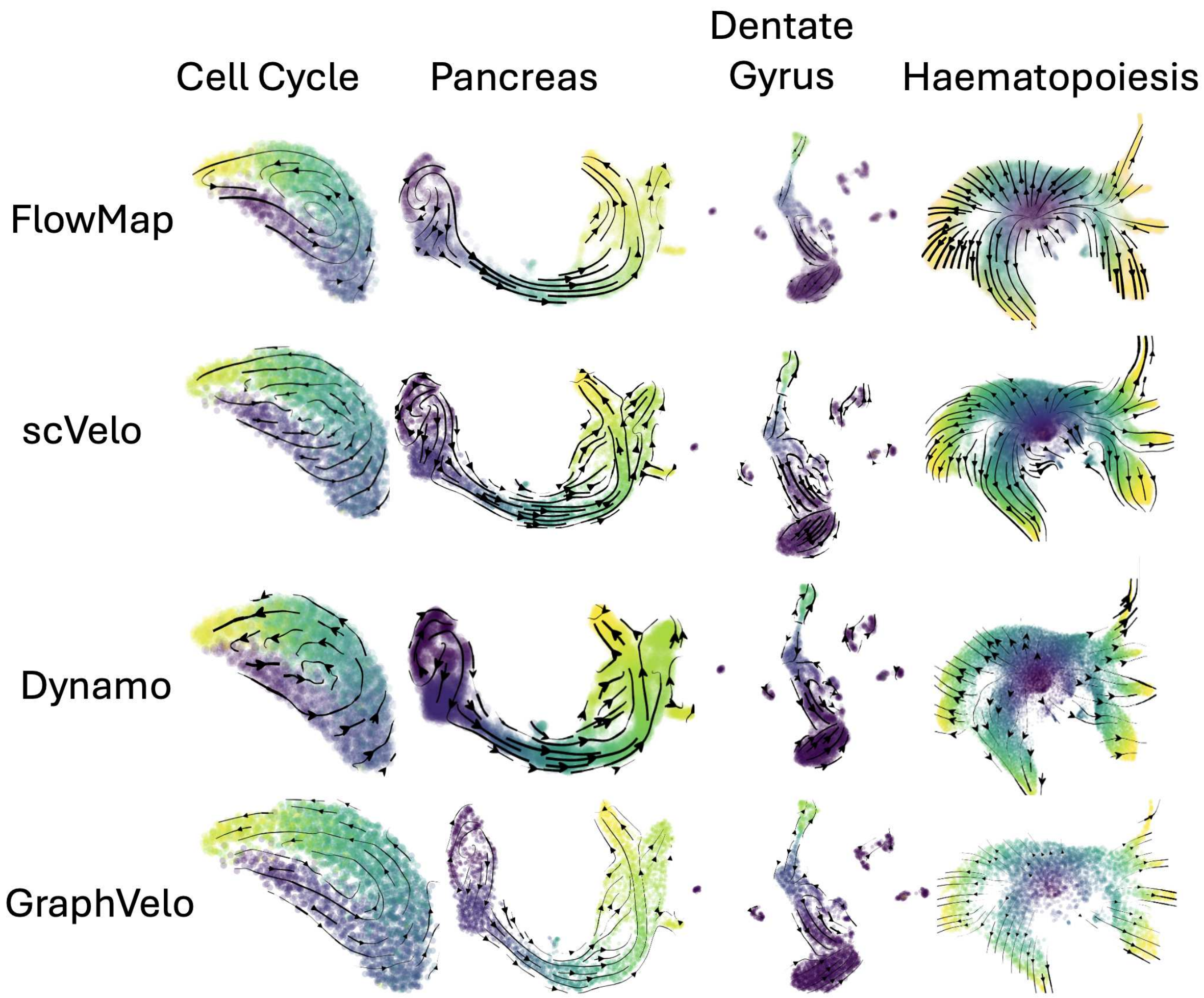

Figure S9: **Benchmark of velocity field reconstruction on real single-cell datasets.** Low-dimensional embeddings with overlaid RNA velocity fields for representative datasets exhibiting diverse dynamical structures, including continuous, curved, and branching trajectories. Each row corresponds to a different method or condition, illustrating variations in the coherence and structure of inferred velocity fields across embeddings.

bioRxiv preprint doi: https://doi.org/10.64898/2026.09.21.753192; this version posted September 24, 2026. The copyright holder for this preprint (which was not certified by peer review) is the author/funder, who has granted bioRxiv a license to display the preprint in perpetuity. It is made available under a CC-BY 4.0 International license.

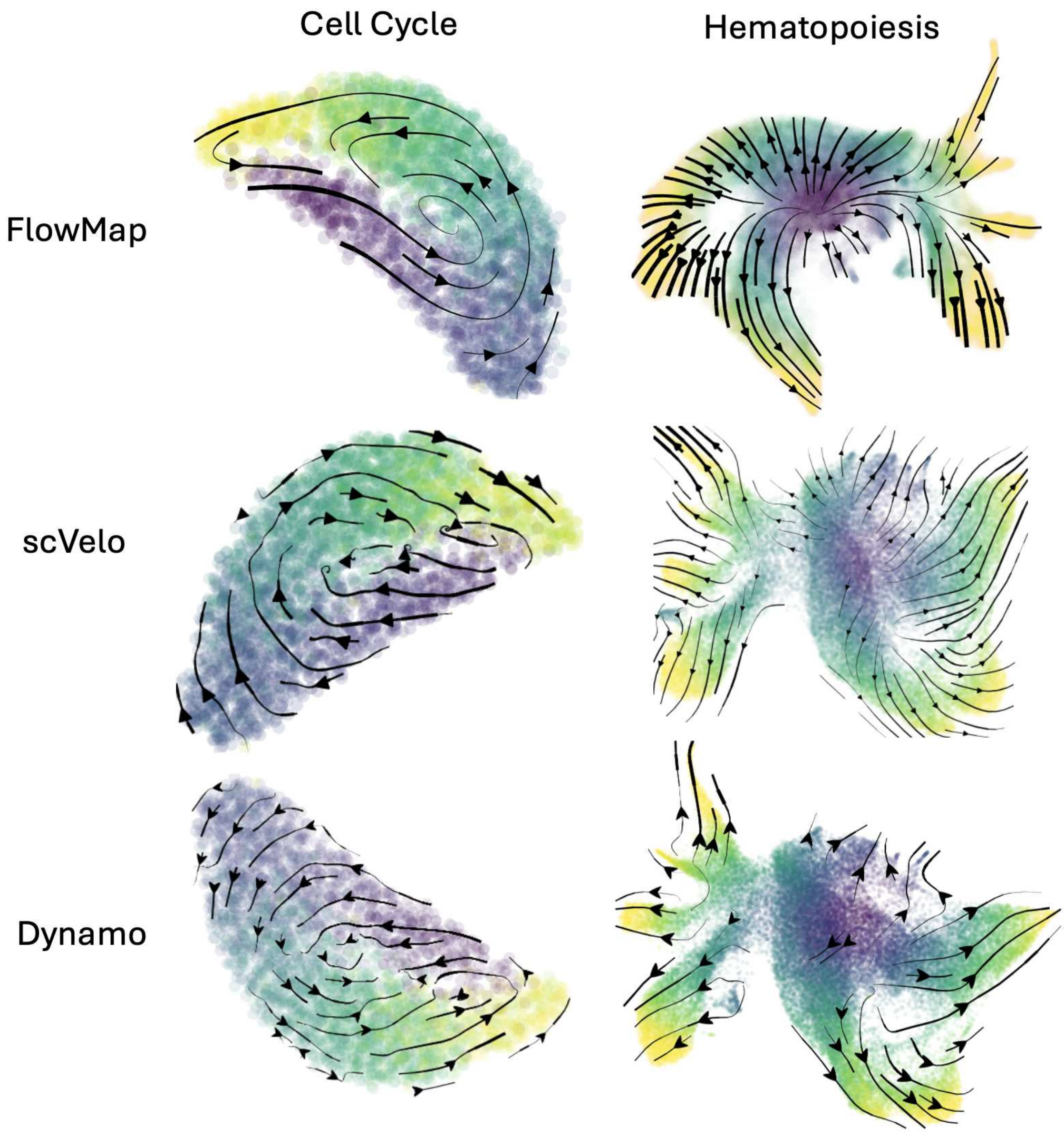

Figure S10: **Benchmark of complete embedding and velocity analysis pipelines.** Comparison of embeddings between FlowMap, scVelo and Dynamo on the cell-cycle and hematopoiesis datasets. The embedding is made with the default UMAP parameters.

bioRxiv preprint doi: https://doi.org/10.64898/2026.09.21.753192; this version posted September 24, 2026. The copyright holder for this preprint (which was not certified by peer review) is the author/funder, who has granted bioRxiv a license to display the preprint in perpetuity. It is made available under a CC-BY 4.0 International license.

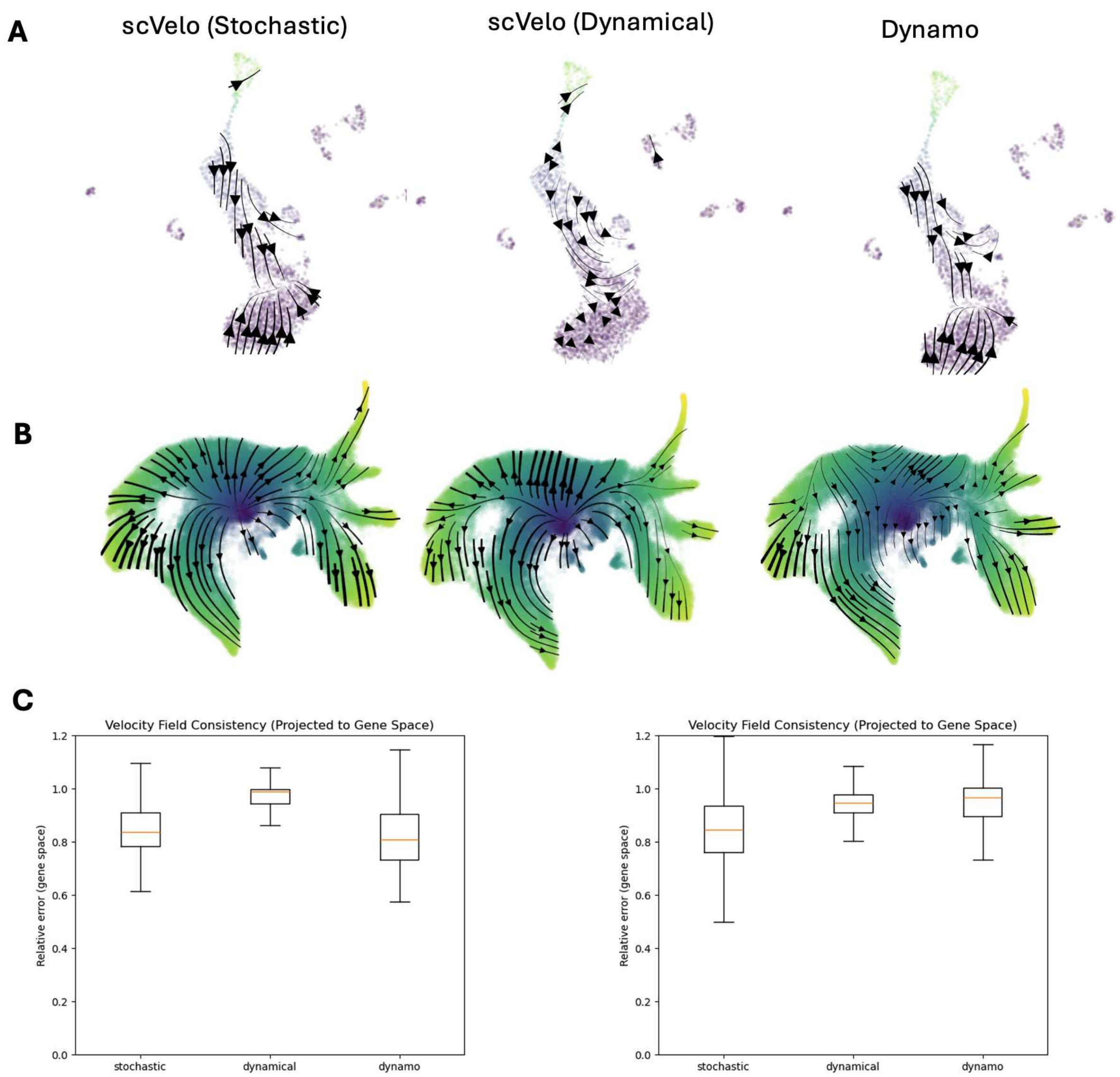

Figure S11: **Comparison of velocity estimation methods.**
(A) Velocity vectors inferred by scVelo (stochastic), scVelo (dynamical), and Dynamo on the Dentate Gyrus dataset, visualized on a common embedding estimated using scVelo. (B) Velocity vectors inferred by scVelo (stochastic), scVelo (dynamical), and Dynamo on the Larry dataset, visualized on a common embedding estimated using FlowMap. (C) Quantitative evaluation of velocity field consistency after projecting the learned low-dimensional vector fields back to gene space. Box plots show cell-level relative errors between predicted and observed velocities for the Dentate Gyrus dataset (left) and Larry dataset (right).

bioRxiv preprint doi: https://doi.org/10.64898/2026.09.21.753192; this version posted September 24, 2026. The copyright holder for this preprint (which was not certified by peer review) is the author/funder, who has granted bioRxiv a license to display the preprint in perpetuity. It is made available under a CC-BY 4.0 International license.

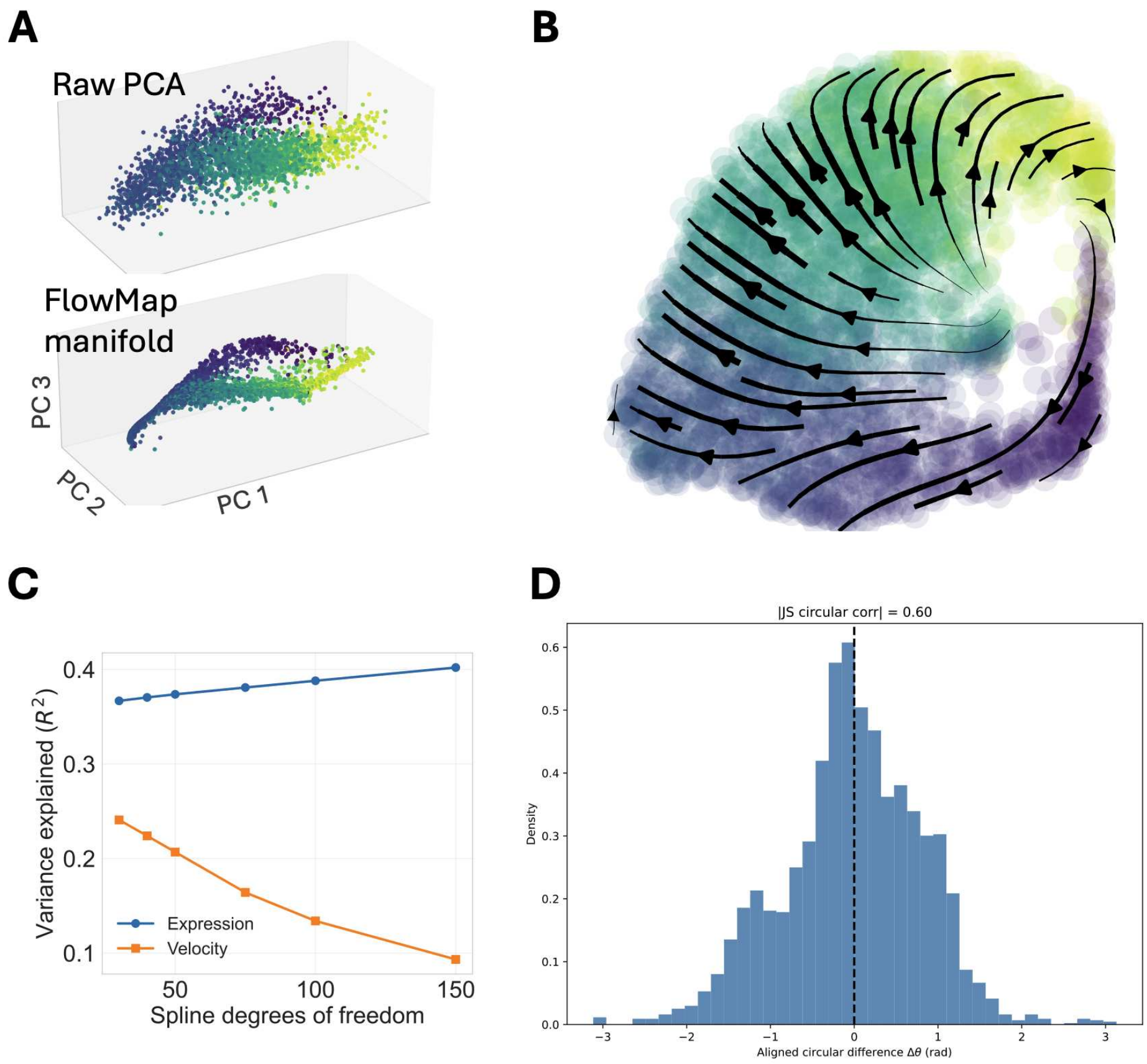

Figure S12: **Diagnostics of FlowMap embedding using all highly variable genes.** (A) FlowMap embedding and inferred RNA velocity field constructed using all 816 highly variable genes in the RPE1–FUCCI scEU-seq dataset. (B) Reconstruction performance as a function of spline complexity, showing variance explained (R^2) for gene expression and RNA velocity across different degrees of freedom. (C) Evaluation of embedding geometry against ground-truth cell-cycle position using circular differences. Circular differences between embedding-derived angles and ground-truth phase are computed after correcting for a global rotational offset, and alignment is summarized using the Jammalamadaka–Sengupta circular–circular correlation coefficient.

bioRxiv preprint doi: https://doi.org/10.64898/2026.09.21.753192; this version posted September 24, 2026. The copyright holder for this preprint (which was not certified by peer review) is the author/funder, who has granted bioRxiv a license to display the preprint in perpetuity. It is made available under a CC-BY 4.0 International license.

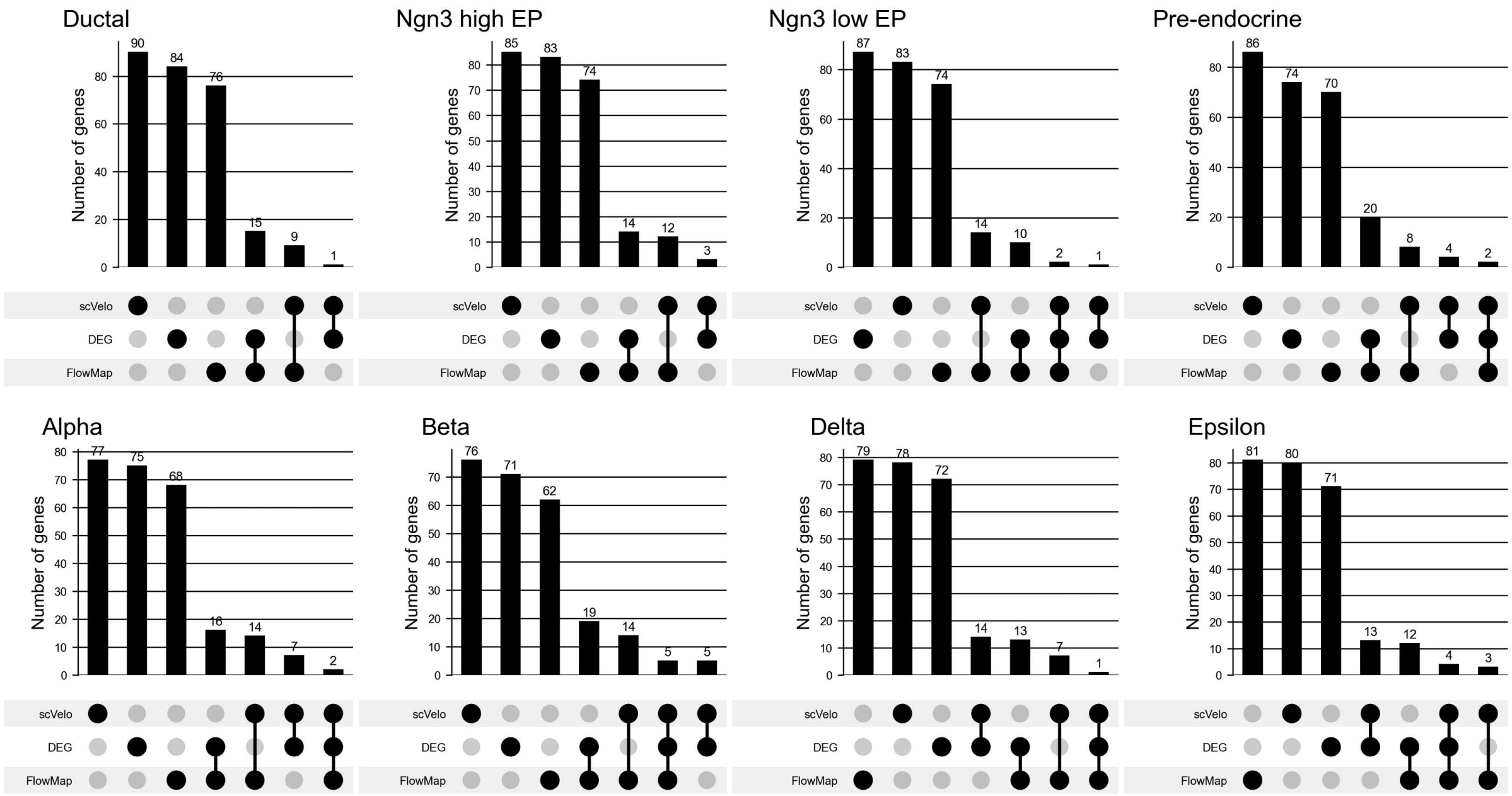

Figure S13: **Overlap of prioritized genes across methods in pancreas endocrinogenesis.** UpSet plots showing the overlap among the top 100 genes prioritized by FlowMap, differentially expressed genes (DEGs), and scVelo differential kinetics for each pancreatic cell state. Gene sets were computed independently within each cell type.

bioRxiv preprint doi: https://doi.org/10.64898/2026.09.21.753192; this version posted September 24, 2026. The copyright holder for this preprint (which was not certified by peer review) is the author/funder, who has granted bioRxiv a license to display the preprint in perpetuity. It is made available under a CC-BY 4.0 International license.

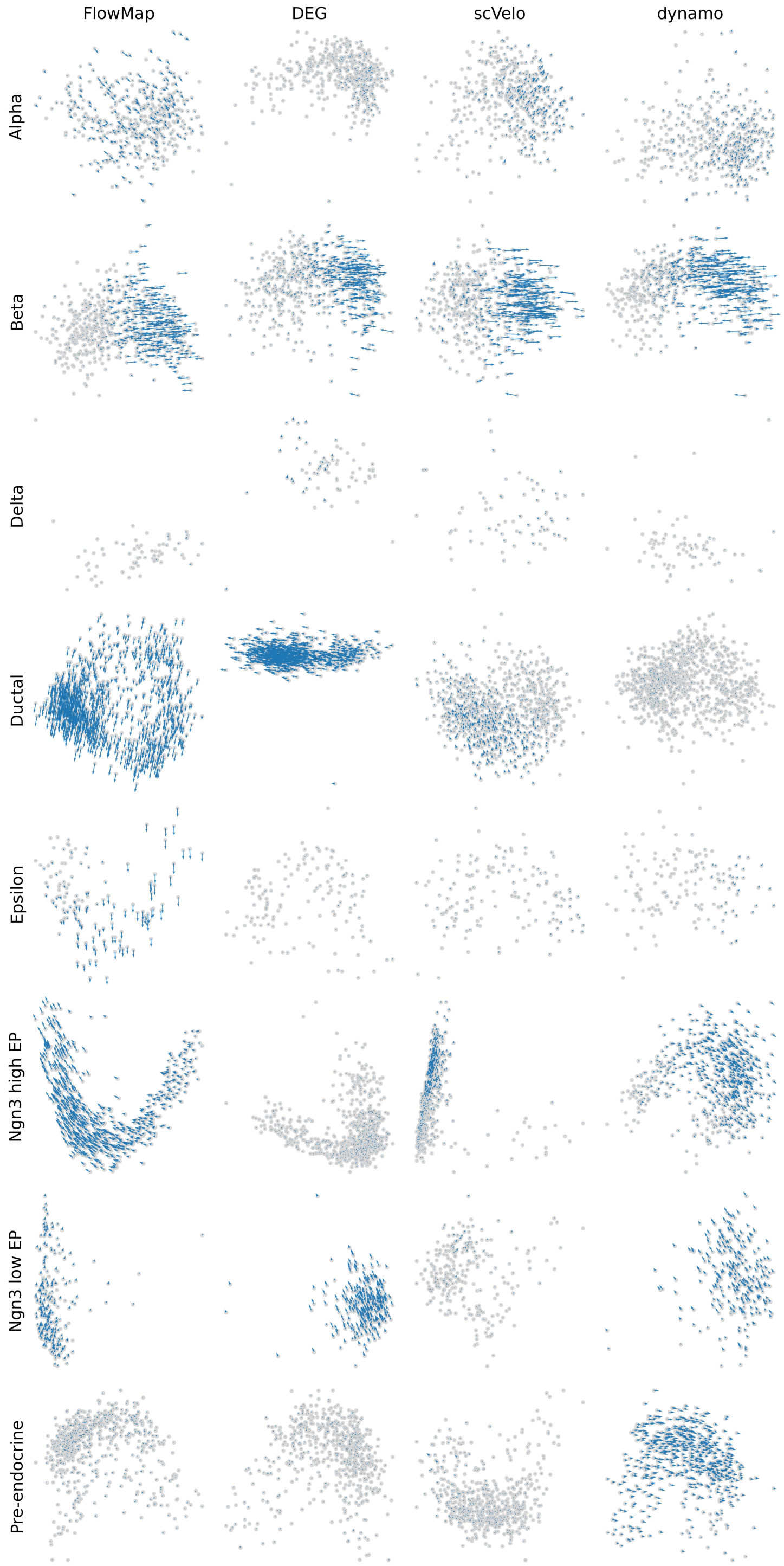

Figure S14: Local velocity structure induced by method-specific prioritized genes. Principal component projections of pancreatic cell states using the top 100 genes prioritized by each method within each cell type. For each panel, PCA was computed using only the selected genes from the corresponding method and cell type, and RNA velocity vectors were projected into the resulting two-dimensional PC space.

bioRxiv preprint doi: https://doi.org/10.64898/2026.09.21.753192; this version posted September 24, 2026. The copyright holder for this preprint (which was not certified by peer review) is the author/funder, who has granted bioRxiv a license to display the preprint in perpetuity. It is made available under a CC-BY 4.0 International license.

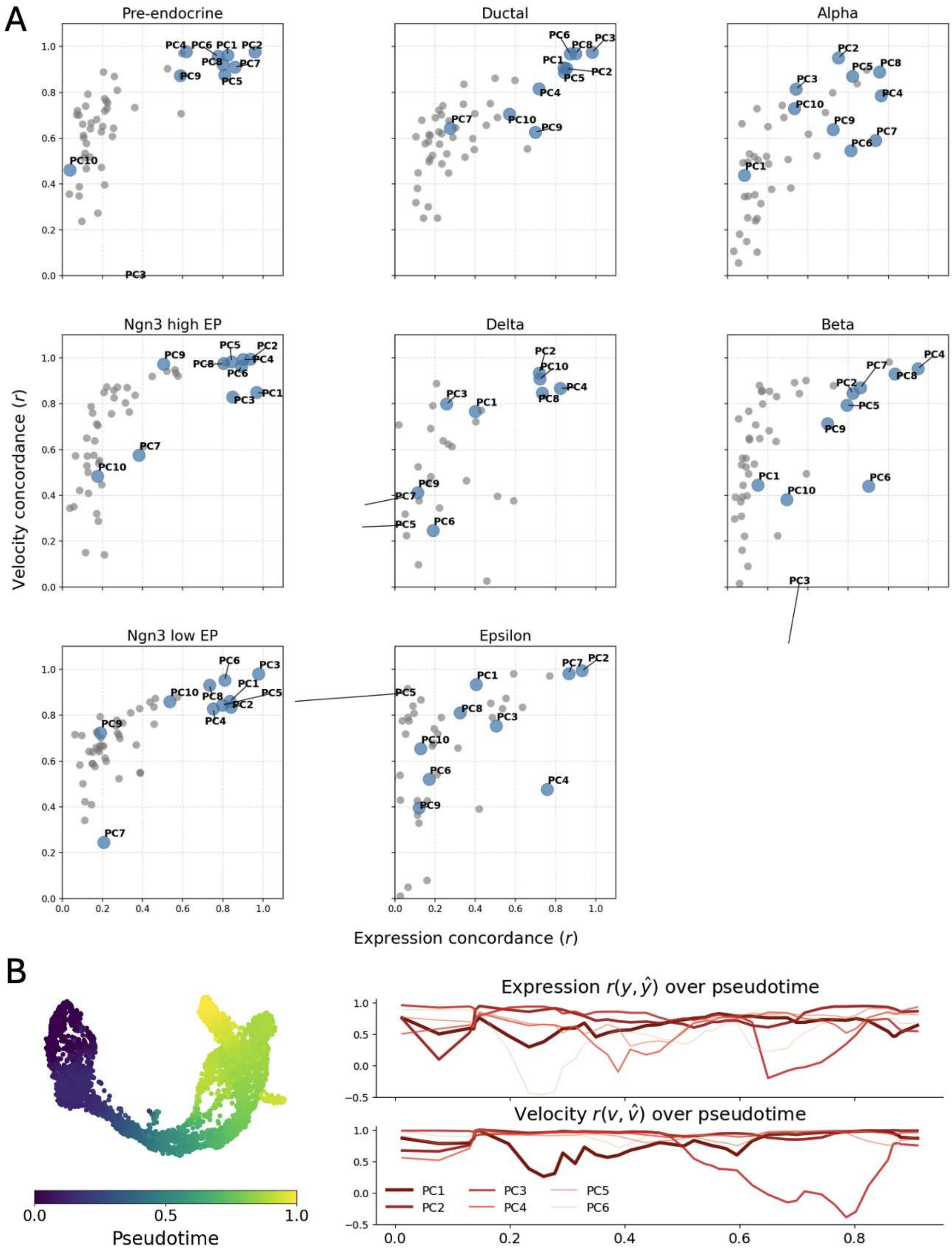

Figure S15: **PC-level reconstruction concordance across pancreatic cell types.**
(A) For each cell type, expression and RNA velocity reconstruction consistencies are shown for PCs 1–50, with each point representing one principal component. PCs 1–10 are highlighted and labeled.
(B) Expression and RNA velocity reconstruction consistency across pseudotime for PCs 1–6.

bioRxiv preprint doi: https://doi.org/10.64898/2026.09.21.753192; this version posted September 24, 2026. The copyright holder for this preprint (which was not certified by peer review) is the author/funder, who has granted bioRxiv a license to display the preprint in perpetuity. It is made available under a CC-BY 4.0 International license.

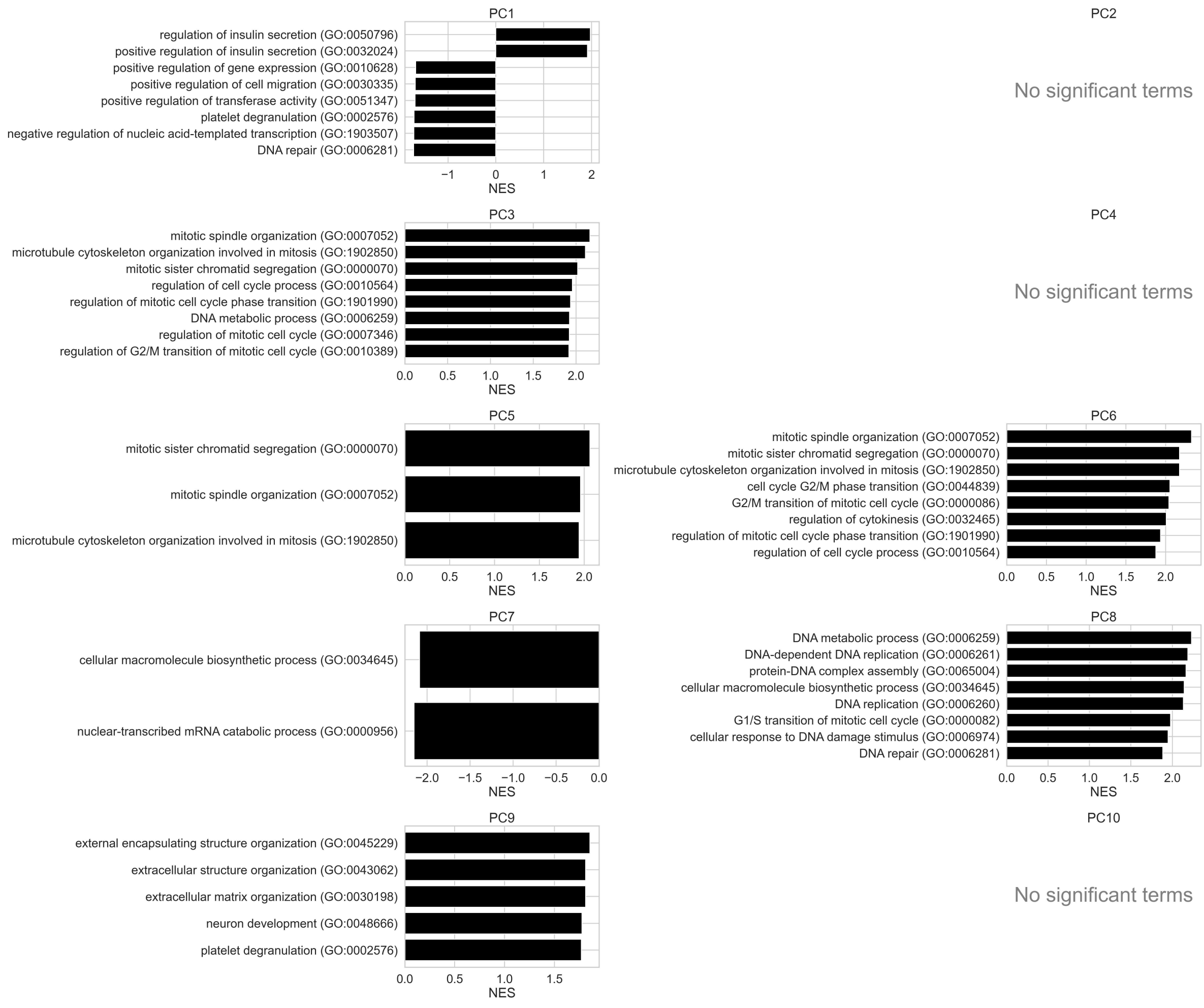

Figure S16: **Gene set enrichment analysis of principal components in pancreatic differentiation.** Preranked GSEA was performed on gene loadings for PCs 1–10 using GO Biological Process gene sets. Each panel shows the top enriched terms for a given PC when significant (FDR < 0.05); PCs without significant enrichment are indicated accordingly.

bioRxiv preprint doi: https://doi.org/10.64898/2026.09.21.753192; this version posted September 24, 2026. The copyright holder for this preprint (which was not certified by peer review) is the author/funder, who has granted bioRxiv a license to display the preprint in perpetuity. It is made available under a CC-BY 4.0 International license.

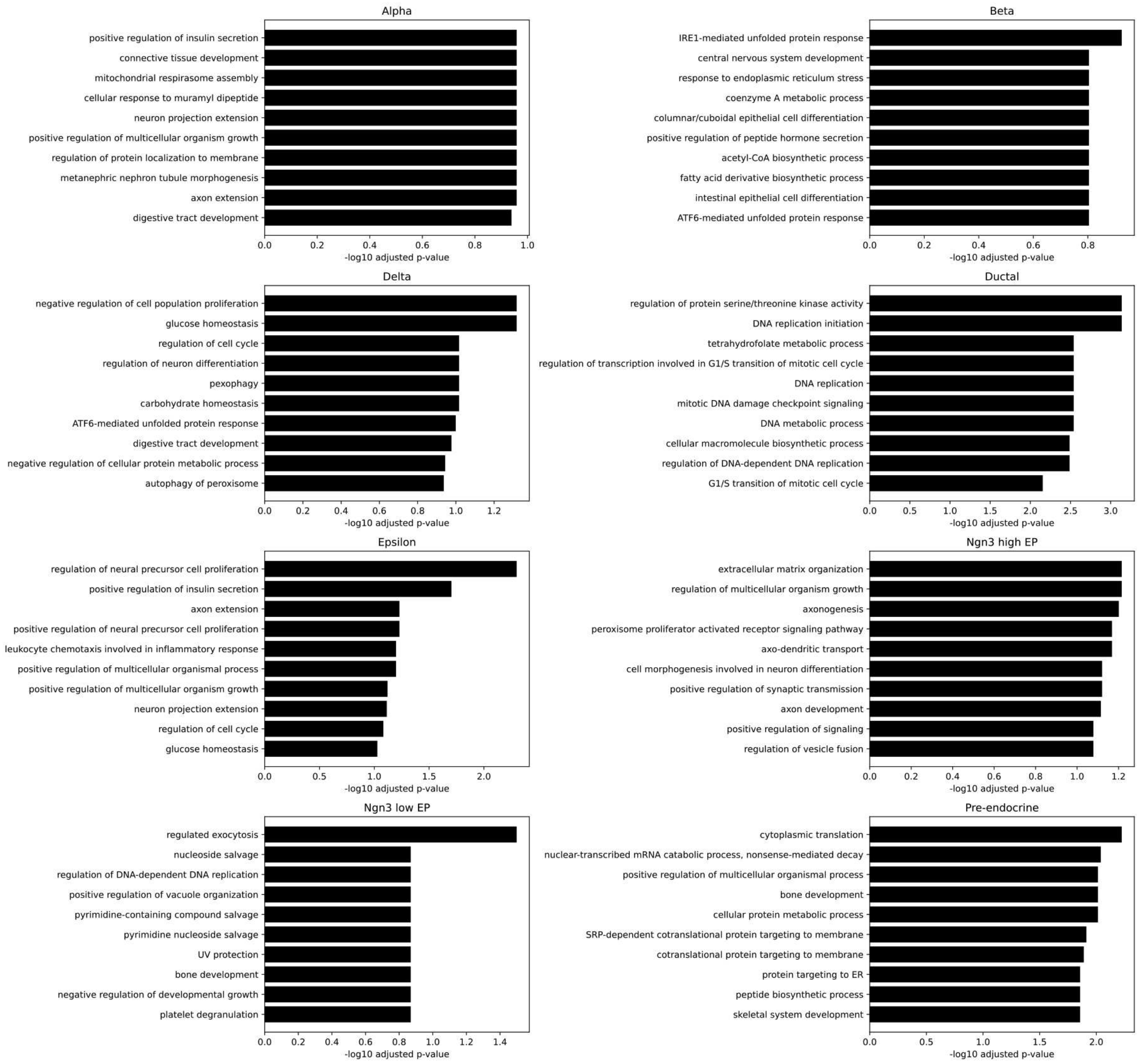

Figure S17: **GO enrichment of FlowMap-prioritized pancreatic genes.** Top GO biological process terms enriched among the top 100 FlowMap-prioritized genes for each pancreatic cell type.

bioRxiv preprint doi: https://doi.org/10.64898/2026.09.21.753192; this version posted September 24, 2026. The copyright holder for this preprint (which was not certified by peer review) is the author/funder, who has granted bioRxiv a license to display the preprint in perpetuity. It is made available under a CC-BY 4.0 International license.

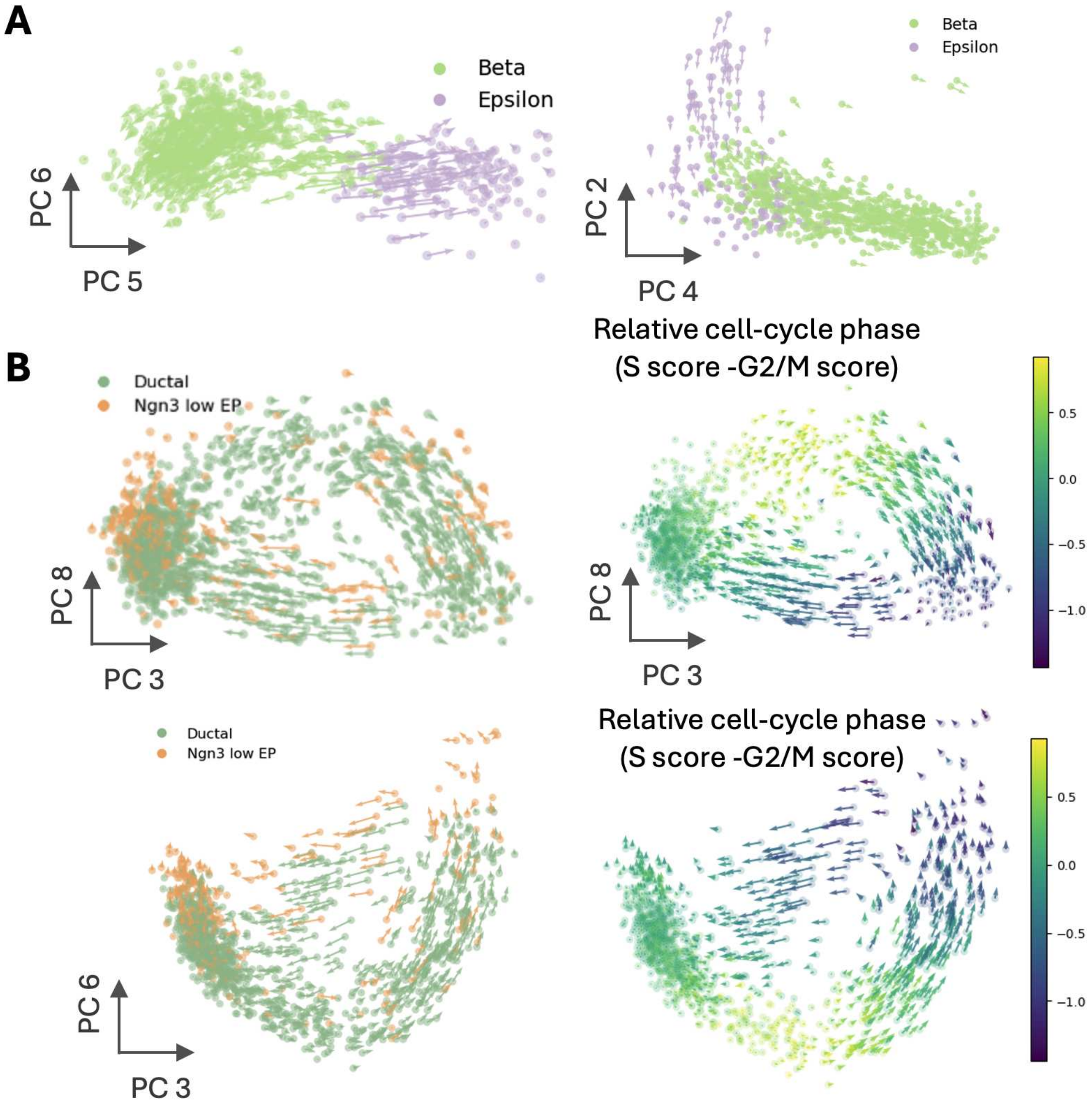

Figure S18: **Additional vector field projections in pancreatic differentiation.** Velocity fields projected onto additional principal component subspaces for pancreatic cell populations, highlighting cell-type–specific geometric structure across multiple latent dimensions.

bioRxiv preprint doi: https://doi.org/10.64898/2026.09.21.753192; this version posted September 24, 2026. The copyright holder for this preprint (which was not certified by peer review) is the author/funder, who has granted bioRxiv a license to display the preprint in perpetuity. It is made available under a CC-BY 4.0 International license.

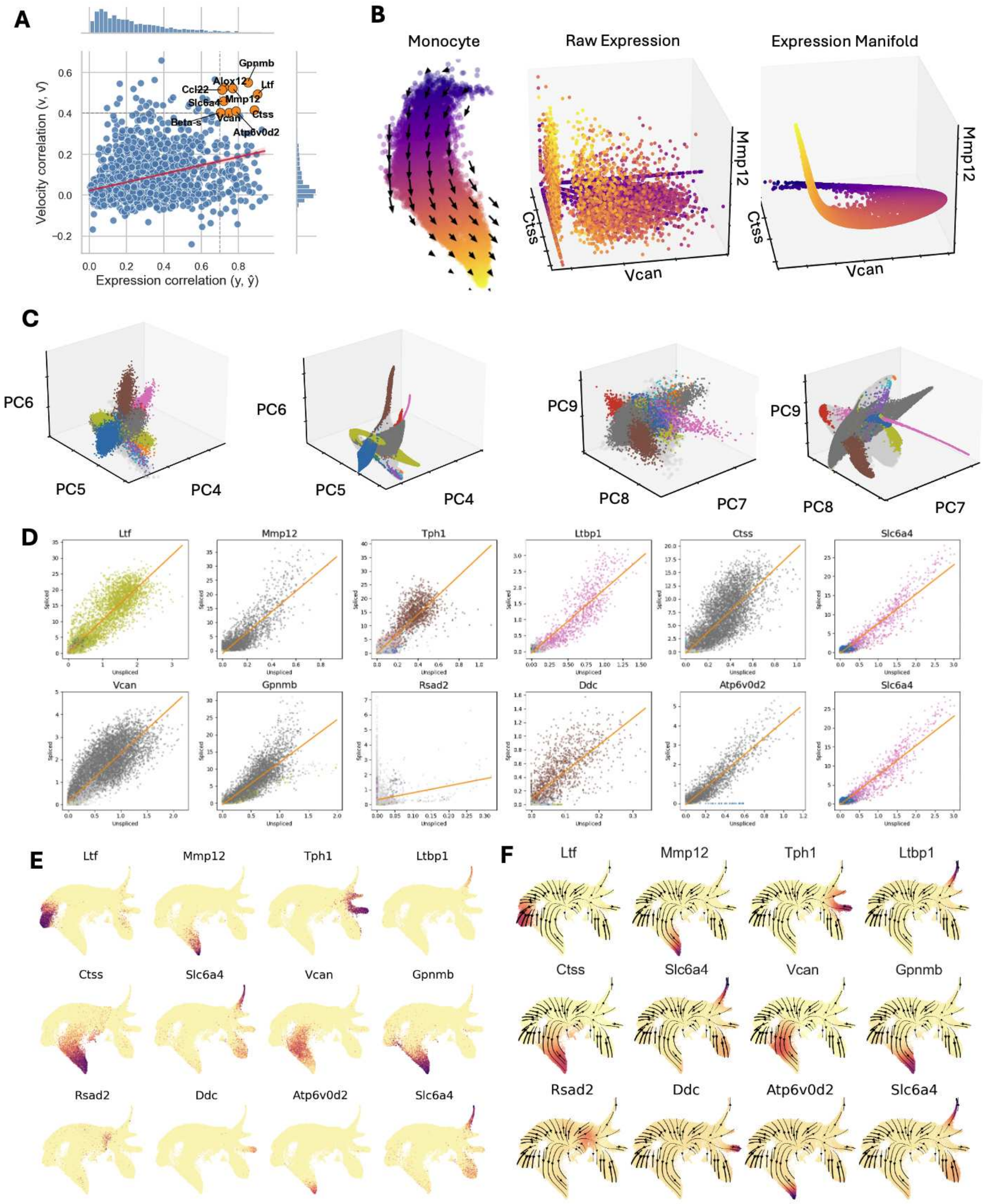

Figure S19: **Gene-level velocity correlations and expression structure on the embedding.** (**A**) Scatter plot showing gene-wise correlation between expression similarity and velocity similarity across cells, with selected genes highlighted. (**B**) Monocyte embedding with velocity vectors, alongside raw gene expression and the corresponding expression manifold for representative genes. (**C**) Principal component representations of gene expression, shown in raw PC space and after mapping to the learned manifold. (**D**) Phase portraits for selected genes, showing spliced versus unspliced transcript counts across cells with linear reference fits. (**E**) Raw gene expression projected onto the embedding for multiple genes. (**F**) Smoothed gene expression projected onto the embedding and visualized together with the learned velocity field, where the expression values can be viewed as a scalar field defined over the embedding.

bioRxiv preprint doi: https://doi.org/10.64898/2026.09.21.753192; this version posted September 24, 2026. The copyright holder for this preprint (which was not certified by peer review) is the author/funder, who has granted bioRxiv a license to display the preprint in perpetuity. It is made available under a CC-BY 4.0 International license.

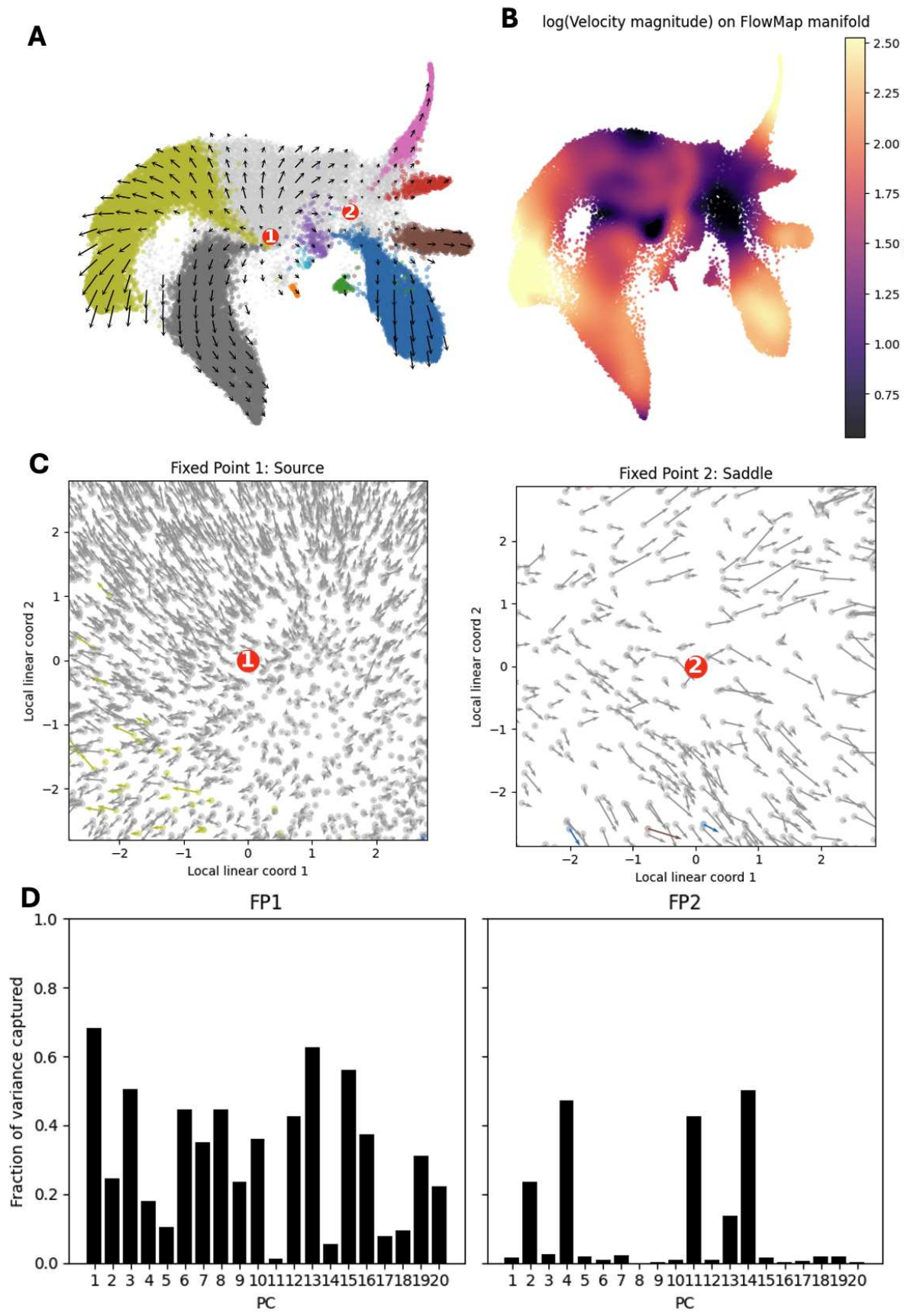

Figure S20: **Fixed point structure and local linear analysis on the FlowMap embedding.** (**A**) FlowMap embedding with the learned velocity field overlaid, highlighting two identified fixed points. (**B**) Visualization of the log-transformed velocity magnitude projected onto the FlowMap embedding. (**C**) Local linearized views of the velocity field around each fixed point, shown in locally flattened coordinates. (**D**) Alignment between principal components and the local linear subspace at each fixed point, quantified by the fraction of variance captured for the top principal components.

bioRxiv preprint doi: https://doi.org/10.64898/2026.09.21.753192; this version posted September 24, 2026. The copyright holder for this preprint (which was not certified by peer review) is the author/funder, who has granted bioRxiv a license to display the preprint in perpetuity. It is made available under a CC-BY 4.0 International license.

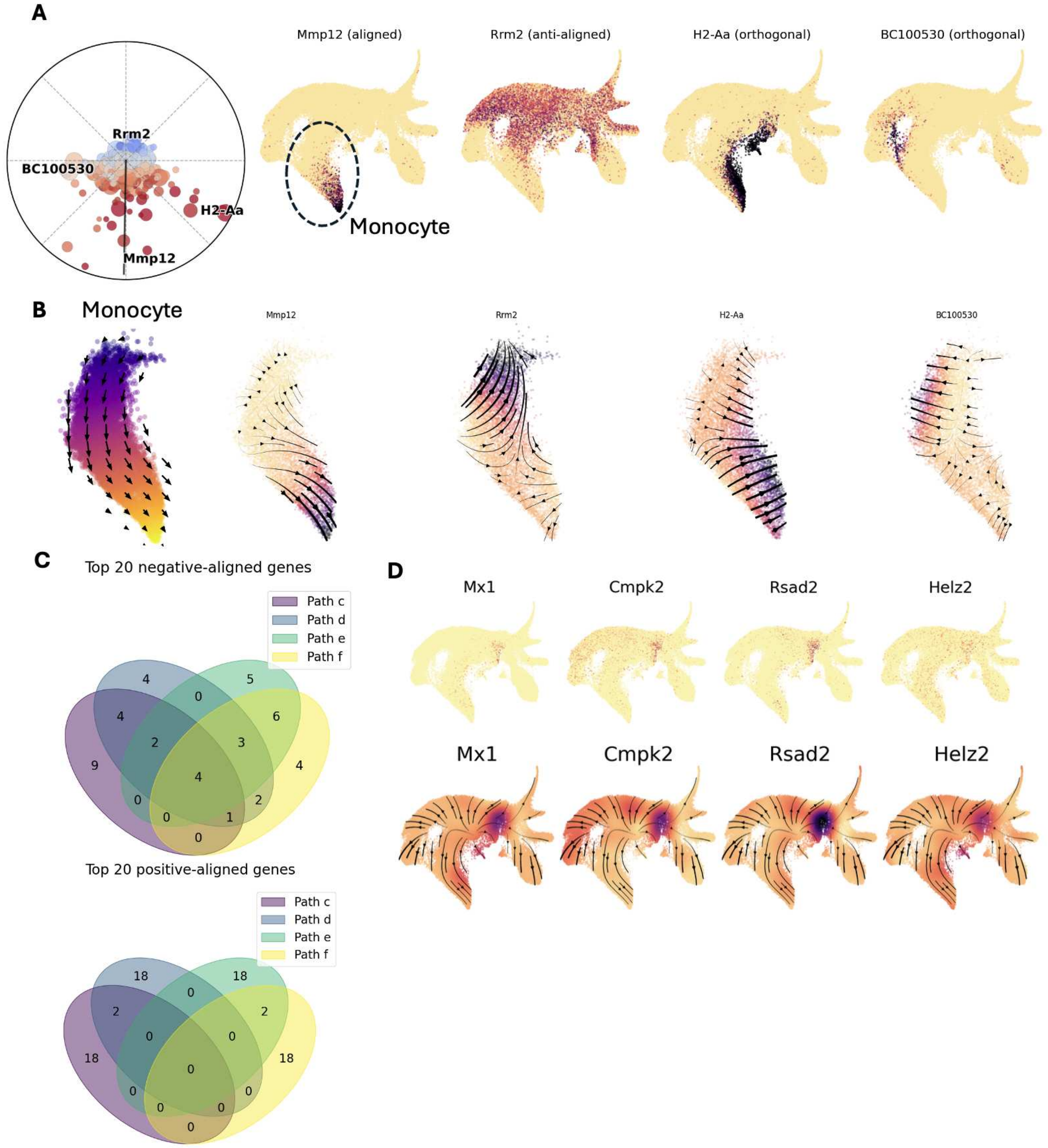

Figure S21: **Gradient alignment and gene expression patterns in the monocyte trajectory.** **(A)** Polar plot showing the alignment between gene-expression gradients and the inferred velocity field within the monocyte trajectory. Each point represents a gene, positioned by the angle between its gradient and the local velocity direction. One representative gene from each alignment category is highlighted, with corresponding smoothed expression patterns shown on the right. **(B)** Gradient vector fields of the same four genes restricted to monocyte cells. Streamlines indicate the direction of increasing gene expression. **(C)** Venn diagrams showing overlap among the top 20 negatively aligned genes (top) and positively aligned genes (bottom) across four inferred differentiation paths. **(D)** Smoothed expression of negatively aligned genes shared across all paths, visualized on the embedding.

bioRxiv preprint doi: https://doi.org/10.64898/2026.09.21.753192; this version posted September 24, 2026. The copyright holder for this preprint (which was not certified by peer review) is the author/funder, who has granted bioRxiv a license to display the preprint in perpetuity. It is made available under a CC-BY 4.0 International license.

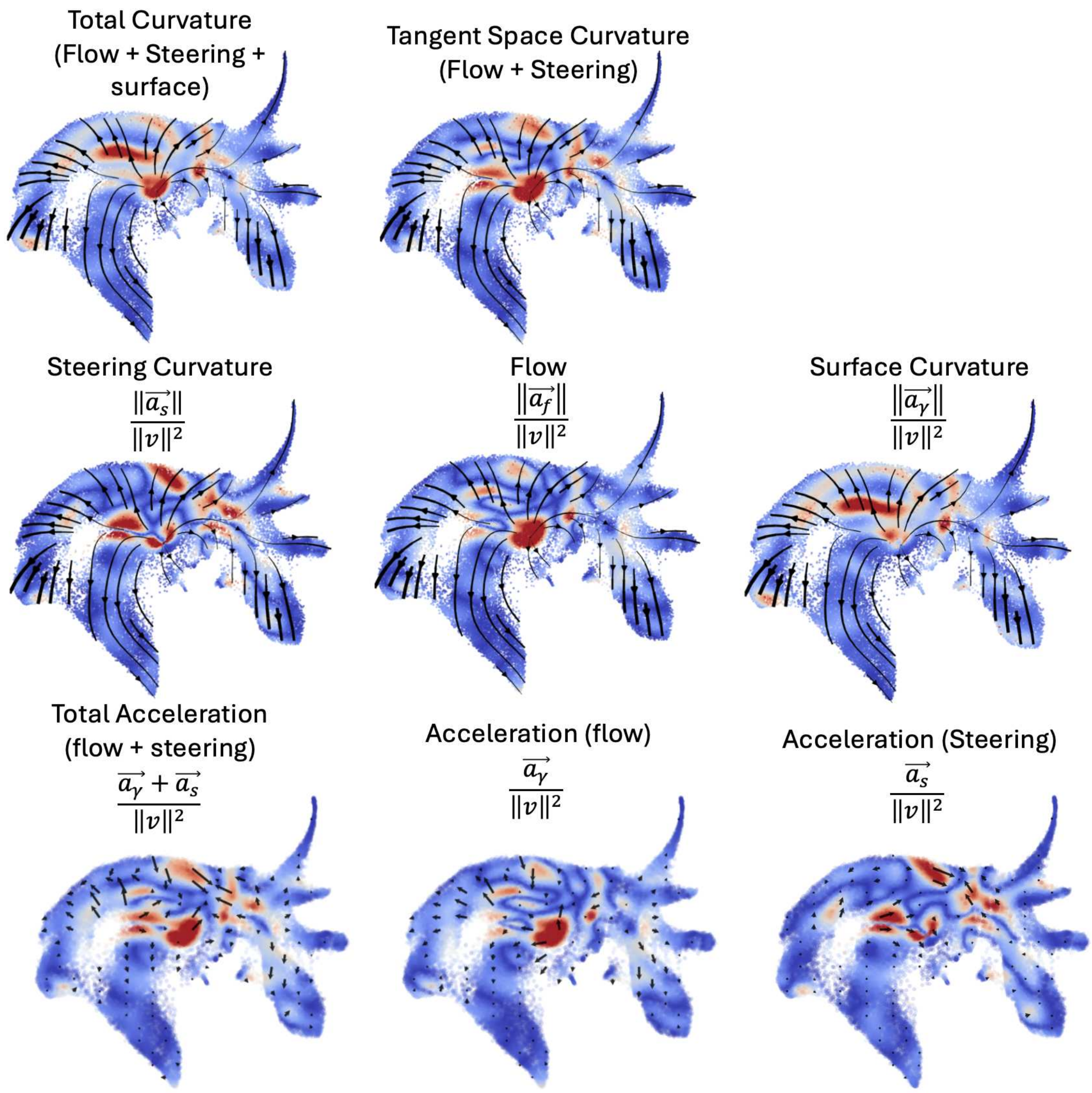

Figure S22: **Additional visualization of curvature and acceleration components on the FlowMap embedding.** Panels show the decomposition of RNA velocity acceleration into flow, steering, and surface components, together with the corresponding curvature quantities across the embedded developmental manifold. Streamlines indicate the inferred velocity field in the first two rows, while colors represent the magnitude of the corresponding acceleration or curvature term. In the third row, acceleration components are visualized directly as quiver vectors.

bioRxiv preprint doi: https://doi.org/10.64898/2026.09.21.753192; this version posted September 24, 2026. The copyright holder for this preprint (which was not certified by peer review) is the author/funder, who has granted bioRxiv a license to display the preprint in perpetuity. It is made available under a CC-BY 4.0 International license.

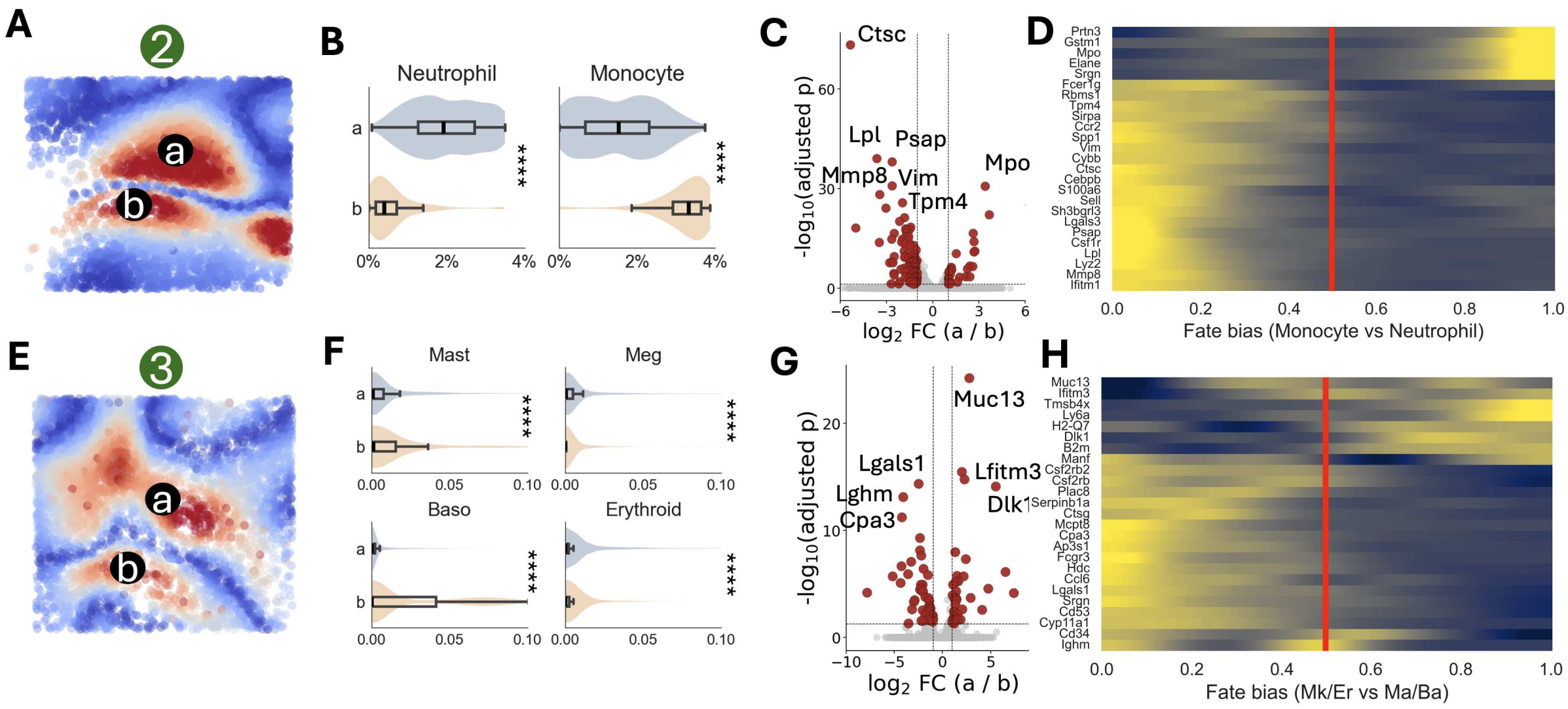

Figure S23: **Additional steering-curvature regions associated with distinct fate biases and transcriptional programs. (A–D)** Analysis of steering-curvature region 2.
(A) FlowMap embedding showing the selected region and two subregions (a and b).
(B) Comparison of inferred fate probabilities between subregions.
(C) Differential expression analysis between subregions a and b.
(D) Expression of differentially expressed genes ordered by relative fate bias within the selected region.
(E–H) Same analysis as in (A–D) for steering-curvature region 3.

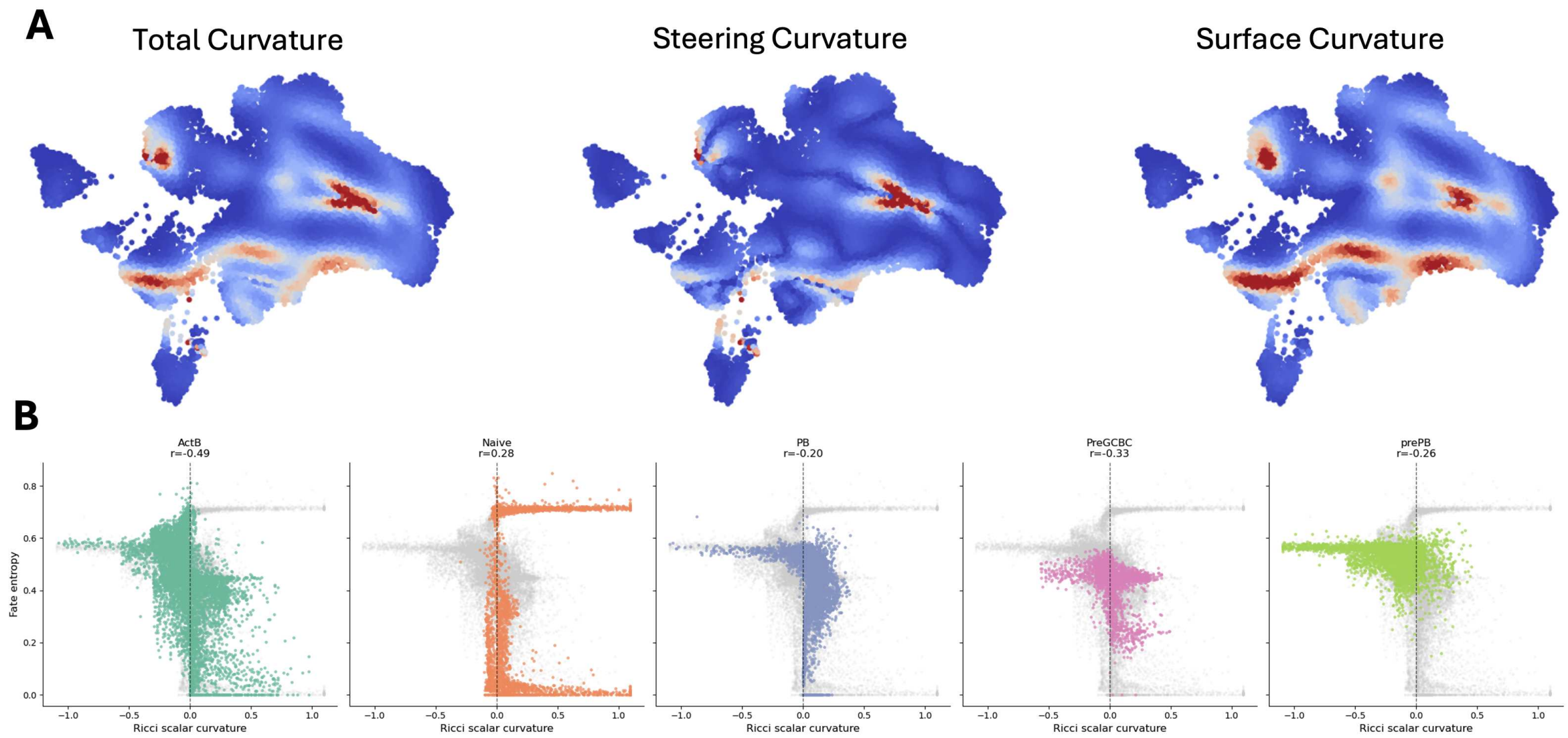

Figure S24: **Curvature structure, gene expression patterns, and fate entropy across B-cell differentiation states.**
(A) Total curvature decomposed into steering and surface curvature components on the FlowMap embedding.
(B) Scatter plots of Ricci scalar curvature versus CellRank fate entropy for individual B-cell populations. Pearson correlation coefficients are indicated above each panel.

bioRxiv preprint doi: https://doi.org/10.64898/2026.09.21.753192; this version posted September 24, 2026. The copyright holder for this preprint (which was not certified by peer review) is the author/funder, who has granted bioRxiv a license to display the preprint in perpetuity. It is made available under a CC-BY 4.0 International license.

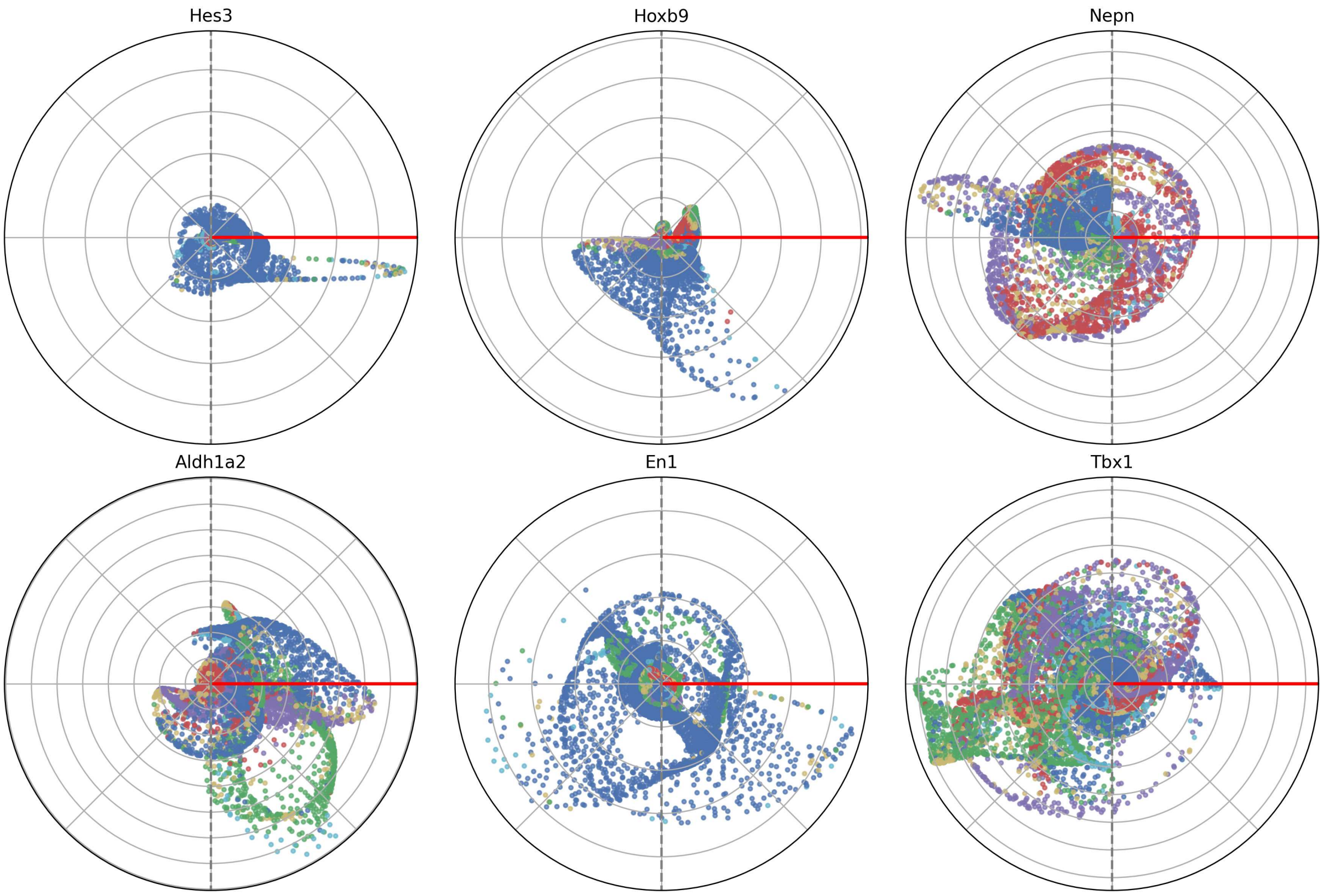

Figure S25: **Gene-specific alignment of expression gradients with RNA velocity for mouse organogenesis data.** Points show per-cell gradient direction (angle) and strength (radius) relative to RNA velocity, colored by cell type.
